# Efficacy of Non-Pharmacological Interventions to Prevent and Treat Delirium in Older Patients: A Systematic Overview. The SENATOR project ONTOP Series

**DOI:** 10.1371/journal.pone.0123090

**Published:** 2015-06-10

**Authors:** Iosief Abraha, Fabiana Trotta, Joseph M. Rimland, Alfonso Cruz-Jentoft, Isabel Lozano-Montoya, Roy L. Soiza, Valentina Pierini, Paolo Dessì Fulgheri, Fabrizia Lattanzio, Denis O’Mahony, Antonio Cherubini

**Affiliations:** 1 Geriatrics and Geriatric Emergency Care, Italian National Research Center on Aging (IRCCS-INRCA), Ancona, Italy; 2 Scientific Direction, Italian National Research Center on Aging (IRCCS-INRCA), Ancona, Italy; 3 Servicio de Geriatría, Hospital Universitario Ramón y Cajal, Madrid, Spain; 4 Department of Medicine for the Elderly, Woodend Hospital, Aberdeen, United Kingdom; 5 Clinica di Medicina Interna e Geriatria, Politecnica University of the Marche Region, Ancona, Italy; 6 Department of Medicine, University College Cork, Cork, Ireland; D'or Institute of Research and Education, BRAZIL

## Abstract

**Background:**

Non-pharmacological intervention (e.g. multidisciplinary interventions, music therapy, bright light therapy, educational interventions etc.) are alternative interventions that can be used in older subjects. There are plenty reviews of non-pharmacological interventions for the prevention and treatment of delirium in older patients and clinicians need a synthesized, methodologically sound document for their decision making.

**Methods and Findings:**

We performed a systematic overview of systematic reviews (SRs) of comparative studies concerning non-pharmacological intervention to treat or prevent delirium in older patients. The PubMed, Cochrane Database of Systematic Reviews, EMBASE, CINHAL, and PsychINFO (April 28th, 2014) were searched for relevant articles. AMSTAR was used to assess the quality of the SRs. The GRADE approach was used to assess the quality of primary studies. The elements of the multicomponent interventions were identified and compared among different studies to explore the possibility of performing a meta-analysis. Risk ratios were estimated using a random-effects model. Twenty-four SRs with 31 primary studies satisfied the inclusion criteria. Based on the AMSTAR criteria twelve reviews resulted of moderate quality and three resulted of high quality. Overall, multicomponent non-pharmacological interventions significantly reduced the incidence of delirium in surgical wards [2 randomized trials (RCTs): relative risk (RR) 0.71, 95% Confidence Interval (CI) 0.59 to 0.86, I^2^=0%; (GRADE evidence: moderate)] and in medical wards [2 CCTs: RR 0.65, 95%CI 0.49 to 0.86, I^2^=0%; (GRADE evidence: moderate)]. There is no evidence supporting the efficacy of non-pharmacological interventions to prevent delirium in low risk populations (i.e. low rate of delirium in the control group)[1 RCT: RR 1.75, 95%CI 0.50 to 6.10 (GRADE evidence: very low)]. For patients who have developed delirium, the available evidence does not support the efficacy of multicomponent non-pharmacological interventions to treat delirium. Among single component interventions only staff education, reorientation protocol (GRADE evidence: very low)] and Geriatric Risk Assessment MedGuide software [hazard ratio 0.42, 95%CI 0.35 to 0.52, (GRADE evidence: moderate)] resulted effective in preventing delirium.

**Conclusions:**

In older patients multi-component non-pharmacological interventions as well as some single-components intervention were effective in preventing delirium but not to treat delirium.

## Introduction

The healthcare system is increasingly demanding rapid access to current research to ensure evidence-based informed decision making and practice. Previously, guideline developers and decision makers were overwhelmed by the number of primary studies; they currently contend with an excess of reviews [1]. The number and variety of systematic reviews (SRs) is rapidly growing. Various sources report that for a single topic several systematic reviews can often be identified [2,3]. Furthermore, there is a tendency to perform systematic reviews of reviews in order to provide clinical decision makers with the evidence they need.

There are several reviews of non-pharmacological interventions for the prevention and treatment of delirium in older patients[4–7]. Delirium is the most common complication of hospital admission in older patients with an incidence rate that varies between 11% and 42% among patients in medical wards[8] and is as high as 80% in some surgical conditions in the post-operative phase[9]. Delirium is associated with increased morbidity, mortality and length of hospital stay as well as increased use of healthcare services and costs[10,11]. Since there is no evidence that pharmacological prevention or treatment of delirium is effective, a great deal of attention has been devoted to non-pharmacological interventions[12–19].

The non-pharmacological interventions to prevent or treat delirium are quite diverse, ranging from simple single component interventions (e.g., music therapy) to complex multicomponent interventions.

This paper describes the methods used to identify all published systematic reviews concerning non-pharmacological interventions for delirium; identifies and critically appraises the primary studies included in the SRs; lists the elements that compose the multicomponent interventions and, based on the components shared among the studies, presents the meta-analyses, critically summarizes the evidence, discusses the limitations and proposes research priorities for future studies.

## Methods

This work is part of the ONTOP (*Optimal Evidence-Based Non-drug Therapies in Older People*) project, a workpackage of a European Union funded FP 7 research named SENATOR (*Software ENgine for the Assessment & Optimization of drug and non-drug Therapy in Older peRsons*). The ONTOP aim is to undertake a literature search of systematic reviews concerning evidence-based non pharmacological treatments of 15 prevalent medical conditions affecting older people, including delirium[20].

To gather the evidence about non-pharmacological intervention to prevent or treat delirium, the ONTOP Evidence Group was established and took responsibility for defining the clinical questions. To define appropriate clinical question the Group identified a list of outcomes and a list of non-pharmacological interventions deemed relevant independent from the evidence according to the Grading of Recommendations Assessment, Development and Evaluation (GRADE) [21]. After three rounds of consultations, important and critical outcomes were identified. For delirium prevention the outcome *delirium incidence* was considered critical whereas for delirium treatment the outcomes *delirium improvement* (intended as either a delirium resolution and a reduction in its severity) and *functional status* (intended as the degree of functional autonomy of the patient) were considered critical. In the present paper only the results of the critical outcomes will be presented. For details see S1 Box, S1 and S2 Tables. For results of secondary outcome see S1 File.

### Search Strategy and Inclusion Criteria for Systematic Reviews

The search sources included Cochrane Database of Systematic Reviews, PubMed, PsychINFO, EMBASE and CINAHL (S2 Box).

Two criteria were considered for further evaluation of an abstract: a) a paper defined as a review or meta-analysis, b) the mention of any non-pharmacological intervention for delirium.

Subsequently, full-texts of relevant abstracts were obtained and screened to identify SRs of interest based on:

1. the use of at least one medical literature database;
2. the inclusion of at least one primary study; and
3. the use of at least one non-pharmacological intervention for delirium prevention or treatment for patients of 60+ years of age.

Only studies written in English, Italian or Spanish were considered.

We assessed the methodological quality of each systematic review using the AMSTAR (A Measurement Tool to Assess Reviews) instrument that contains 11-items to appraise the quality[22]. Two reviewers independently assessed the quality of the SRs and disagreement was resolved by consensus.

### Inclusion Criteria for Primary Studies

From the included SRs we identified any experimental comparative study, either randomized or nonrandomized, that investigated any non-pharmacological intervention to prevent or treat delirium in older patients. Primary studies were excluded if they were observational studies or before-after studies with historical controls.

### Data extraction and management

Extracted data were transferred onto data extraction forms. Information collected included trial characteristics, patients characteristics, intervention and comparator characteristics, and outcome measures.

Pairs of reviewers independently screened titles, abstracts and full-texts of articles. Disagreement was resolved by discussion.

### Itemizing the elements of the multicomponent intervention

To better understand the characteristics of the multicomponent interventions, we itemized each element of the multicomponent intervention. This process helped to decide whether or not it was appropriate to perform a formal meta-analysis of different studies.

### Risk of bias assessment

Assessment of risk of bias for the included primary studies was carried out using criteria from the Cochrane Collaboration[23]. The domains considered were random sequence generation, allocation concealment, blinding of participants and personnel, blinding of outcome assessment, incomplete outcome data, selective reporting and other potential biases (e.g., balance in baseline characteristics). We assigned a risk of bias to one of three categories: low risk, high risk and unclear risk. Two reviewers independently assessed the risk of bias of individual studies and any differences in quality assessment results between raters was resolved through consensus.

### Grading the quality of evidence

The quality of evidence was assessed with GRADE (Grading of Recommendations, Assessment, Development and Evaluation) methodology by a panel of reviewers with experience in geriatrics, internal medicine and methodology.

GRADE assessment took into consideration the risk of bias[24], consistency of results across the available studies[25], precision of the results[26], directness[26], and likelihood of publication bias[27]. Randomized trials were privileged for the GRADE evaluation. Where evidence is without randomized trials the GRADE evaluation is performed for other study designs (e.g., controlled clinical trials). The quality of the evidence was categorized as high, moderate, low, or very low based on the judgments for the primary outcome (delirium incidence for prevention; delirium improvement and functional status for treatment). Summary tables were constructed using GRADEpro version 3.6.

### Data Synthesis and Analysis

Where a meta-analysis was possible with at least two studies, DerSimonian and Laird random-effects models were used to pool risk ratios of delirium incidence (Review Manager software version 5.3). We used the Chi^2^ test and I^2^ statistic to assess heterogeneity[23]. We considered heterogeneity to be statistically significant if the P value is less than 0.1. Publication bias was assessed by visual judgment of a funnel plot and by Egger’s regression method.

## Results

### Systematic reviews

**Fig 1:**
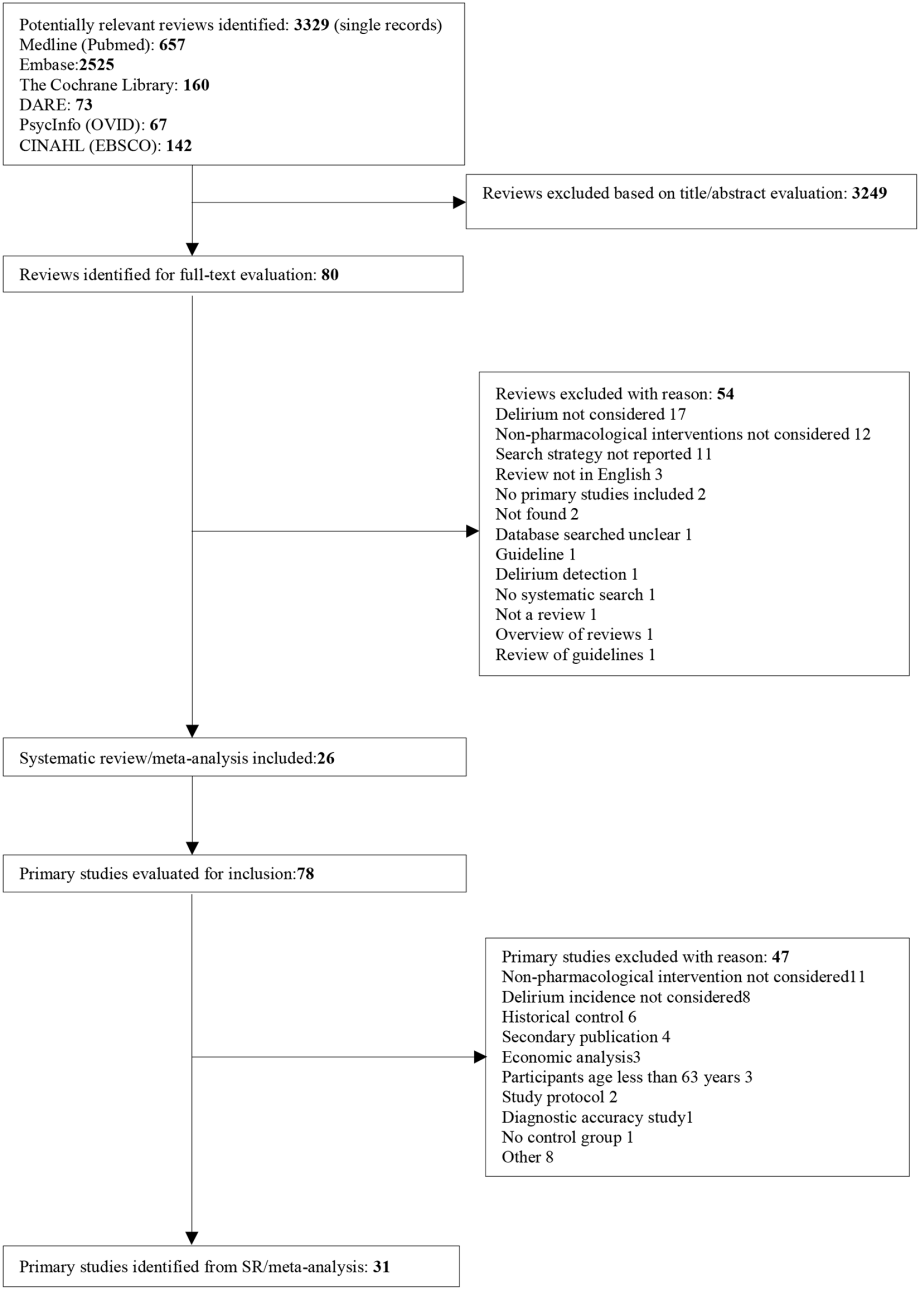
Flow diagram of literature search and study selection.

Our search identified 3329 abstracts after excluding 295 duplicates. Among the 80 potentially relevant publications, 26 were considered relevant for inclusion and 54 were excluded (see Fig 1 for study screening process and S3 Table for list of excluded reviews with reasons). The publication year ranged from 1996 to 2014 and 5 reviews were published in 2014.

While all articles used PubMed to search for primary studies, 13 papers also employed CINAHL[5–7,28–37], 11articles also used the Cochrane Library[7,30,31,34,36,38–43], 10 studies also accessed Embase [7,32,34,36,38,41,43–46], and 3 studies also searched PsychINFO[5,32,36].

None of the SRs was sponsored by a company. Six studies were funded by a governmental institution[7,38,41] or a non-profit organization[5,43,47].

The reviews were heterogeneous. Some reviews, in addition to examining interventions to treat or prevent delirium, also evaluated the pathogenesis of delirium[47], examined the role of sitters[48], or studied approaches to diagnose delirium[28,39,49]. Only two reviews assessed single component interventions (music therapy[33] and earplugs[50]). The remaining SRs appraised multicomponent interventions.

Three SRs ranked as being of high quality (scoring 8–11), 12 of medium quality (scoring 4–7), and 11 of low quality (scoring 0–3) (S4 Table).

Table 1 summarizes the basic characteristics of the SRs.

**Table 1:** Characteristics of Included Systematic Reviews/Meta-analyses.

Systematic review	Aim	Search strategy date	Population	Intervention	Outcomes	Primary studies on non-pharmacological intervention included/total studies included in the review	Reviews also interested in pharmacological intervention
**Alway 2013[51]**	To summarize the evidence of earplugs and eye masks.	Unclear	Critically ill adults	Earplugs and eye masks	Sleep and delirium outcomes	2/7	No
**Bitsch 2004 [47]**	To summarize the pathogenesis of postoperative delirium and to identify strategies for prevention and management.	March 2003	Patients with hip fracture	Educational staff, multicomponent intervention, multidisciplinary team	Delirium prevention and treatment	2/12	Yes
**Carr 2013[52]**	To describe the usage, training, clinical and cost effectiveness of sitters in delirium	October 2011	Heterogeneous population including patients at risk of delirium	Use of sitter	Unclear	4/37	No
**Clegg 2014[53]**	To assess the effectiveness of interventions for preventing delirium	April 2013	People (aged 65+ years) in permanent long-term care residence	Multi- or single-component interventions	Delirium prevention	2/2	Yes
**Cole 1996[6]**	To determine the effectiveness of interventions to prevent delirium.	May 1995	Hospitalized patients	Educational staff, multicomponent intervention, Multidisciplinary team	Delirium prevention	2/10	Yes
**Cole 1998[29]**	To gather evidence about treatment prevention and outcome of delirium.	March 1998	Not specified (any)	Educational staff, multicomponent intervention, Multidisciplinary team	Delirium prevention and treatment	2/15	Yes
**Cole 1999[28]**	To review evidence related to the effectiveness of systematic interventions in preventing or detecting and treating delirium.	March 1998	Any	Educational staff, multicomponent intervention, multidisciplinary team	Delirium prevention and treatment	2/17	No
**Conn 2001[49]**	To outline current approaches to diagnosing and managing delirium in the elderly	1998	Unclear	Family support, multicomponent intervention	Delirium prevention and treatment	1/10	Yes
**Fick 2002[4]**	To assess prevalence, associated features, outcomes, and management of delirium superimposed on dementia.	February 2002	Patients with dementia	Multicomponent intervention	Delirium prevention and treatment	2/7	Yes
**Fox 2012[7]**	To compare the effectiveness of acute geriatric unit care in the acute phase of illness or injury	Unclear (2012?)	Acutely ill or injured adults	Multicomponent intervention	Falls, pressure ulcers, delirium, functional decline, hospital stay, discharge destination, mortality, costs, hospital readmissions	2/19	No
**Gonzales 2003[54]**	To assess prevalence, etiology, prognostic factors, diagnosis and management of delirium	Unclear	Any	Multicomponent intervention	Delirium treatment and prevention	1/unclear	Yes
**Greer 2011[5]**	To assess prevalence, diagnosis and treatment of delirium	November 2010	Adult inpatients	Educational staff, multicomponent intervention, Multidisciplinary team	Delirium incidence.	11/40	Yes
**Grigoryan 2014[55]**	To determine if ortho-geriatric collaboration models improve outcomes	July 2012	Patients with hip fracture	Ortho-geriatric consultation,	In-hospital mortality, length of stay, long-term mortality	2/18	No
**Hempenius 2011[30]**	To assess the efficacy of interventions to prevent delirium and to explore which factors increase the effectiveness of these interventions	July 2009	Patients at risk of delirium	Educational staff, multicomponent intervention, Multidisciplinary team	Delirium incidence	4/16	Yes
**Holroyd-Leduc 2010[38]**	To assess the effective interventions for prevention and treatment of delirium	October 2007	Patients aged 65 or older with delirium or at risk of developing delirium	Educational staff, multicomponent intervention	Delirium prevention and treatment	6/11	Yes
**Inouye 2014[56]**	To provide an overview of epidemiology, causes, and non-pharmacological and pharmacological management of delirium	August 2012	Any population	Pharmacological and non-pharmacological	Delirium prevention and treatment	13/29	Yes
**Mak 2010[34]**	To update evidence-based guidelines for the treatment of proximal femoral fractures	June 2008	Patients with proximal femoral fractures.	Time to surgery, thromboprophylaxis, anaesthesia, analgesia, prophylactic antibiotics, surgical fixation of fractures, nutritional status, mobilization, rehabilitation and daily proactive geriatrics consultation	Surgical wound closure, management of postoperative delirium, osteoporosis treatment and hip protectors	1/128	Yes
**Marik 2006 [44]**	To review the effect of an aging society on the utilization of critical care services and the physiology of aging as it applies to critical illness and prognosis and management issues in the intensive care unit	unclear	Older patients admitted in intensive care unit.	Daily proactive geriatrics consultation, bright light therapy, music therapy	Several outcomes of elderly patients admitted to intensive care unit including prevention of delirium	2/5	Yes
**Milisen 2005[57]**	To determine the characteristics and efficacy of multicomponent intervention strategies for delirium	August 2003	Hospitalized older people	Educational staff, multicomponent intervention	Incidence, duration and severity of delirium, change in cognitive functioning, functional rehabilitation, length of stay and mortality.	7/7	No
**Morrison 1998[58]**	To review the evidence for clinical decisions that medical consultants make for patients with hip fracture and to develop recommendations for care	June 1997	patients with hip fracture	Supportive reorientation and environmental manipulation	Prevention of delirium	1/9	No
**Moyce 2014[59]**	To determine the efficacy of peri-operative interventions in decreasing the incidence of postoperative delirium.	January, March, August 2012	Patients receiving non-cardiac surgery	Any	Incidence of delirium within seven days of surgery	5/29	Yes
**Reston 2012[60]**	To evaluate the effectiveness and safety of in-facility multi-component delirium prevention programs	September 2012	Patients at high risk of developing delirium	Multicomponent programs	Incidence of delirium	13/19	No
**Siddiqi 2007[32]**	To assess the effectiveness of interventions designed to prevent delirium	September 2006	Hospitalized patients	Educational staff, multicomponent intervention	Incidence, duration and severity delirium.	1/6	Yes
**Skingley 2010 [33]**	To identify how music and singing may be used therapeutically by nurses in caring for older people.	Unclear	People 65 years and over with osteoarthritis, delirium, sleep difficulties, chronic obstructive pulmonary disease	Music and singing	pain in patients with osteoarthritis, post-operative delirium prevention, sleep difficulties.	1/1	No
**Weber 2004[39]**	To assess the etiology and risk factors for delirium and to review current strategies for prevention and treatment	unclear	unclear	Multicomponent intervention, multidisciplinary team	Incidence, duration and severity delirium,	4/13	Yes
**Zhang 2013[61]**	To evaluate strategies for preventing post-operative delirium.	August 2012	Adult patients receiving any surgical intervention.	Multiple types of intervention	Incidence, duration and severity delirium,	5/37	Yes

### Primary studies

Overall the 26 SRs yielded 78 primary studies of which 31 satisfied our inclusion criteria. (see Fig 1 and S5 Table for excluded primary studies).

The identified primary studies are described based on the type of the intervention (single or multicomponent), the aim of the intervention (prevention or treatment), the setting (e.g., surgical), and the study design [randomized controlled trial (RCTs), Controlled Clinical Trials (CCT), and Before After (BA) study].

#### Evidence of multicomponent, non-pharmacological interventions to prevent delirium in surgical setting

Nine studies evaluated the efficacy of multicomponent non-pharmacological interventions to prevent delirium in surgical wards. Two studies were randomized trials[62,63], one was a CCT[64] and six were BA studies[65–70]. Except for Chen et al.[66] all studies assessed patients with hip fractures. The study characteristics are described in Table 2. All studies considered the incidence of delirium as primary outcome except Chen et al,[66] who investigated functional and cognitive function as the primary outcomes.

**Table 2:** Characteristics of Primary Studies. Non-Pharmacological Interventions for Delirium Prevention in Surgical Setting.

Author	Type of study	Population	Intervention	Outcome	Study period	Setting	Funding
**Lundstrom 2007[62]**	Randomized trial	199 patients with femoral neck fracture aged 70+ (mean age 82), 74% women	Staff education (focusing on the assessment, prevention and treatment of delirium and associated complication): application of comprehensive geriatric assessment, management and rehabilitation	Primary: number of days of post-operative delirium. Secondary: complications during hospitalization, length of stay, and in-hospital and one-year mortality.	May 2000 and December 2002	A specialized geriatric ward or a conventional orthopedic ward	Government, not for-profit.
**Marcantonio 2001[63]**	Randomized trial	86 patients 65+ admitted emergently for surgical repair of hip fracture (mean age 79), 79% women,	Proactive geriatrics consultation	Primary: delirium incidence (DSI, (MDAS) (CAM) MMSE) Secondary outcomes: delirium severity (MDAS, CAM), cognitive status (MMSE), length of stay, nursing home discharge	not reported	Orthopedic dept.	Private non-profit
**Deschodt 2012[64]**	Controlled clinical trial	171 people with hip fracture aged 65 and older; female 65%	inpatient geriatric consultation teams	Incidence and duration of delirium (CAM), severity of delirium (Delirium Index), and cognitive status (MMSE)	unclear	Two trauma wards	None
**Björkelund 2010[71]**	Before/after study	263 patients with hip fracture, age ≥65 years; female 70%.	Multifactorial intervention (supplemental oxygen, hydration, nutrition, monitoring of vital physiological parameters, adequate pain relief, avoid delay in transfer logistics, daily delirium screening using OBS scale, avoid poly-pharmacy, and perioperative /anesthetic period protocol)	Delirium incidence (SPMSQ; OBS scale)	April 2003–April 2004	Orthopedic ward	Government
**Chen 2011[66]**	Before/after study	256 patients (mean age 71, female 46%) undergoing elective abdominal surgery (e.g. gastrectomy).	The intervention (modified Hospital Elder Life Program): daily hospital-based care protocol, which included 3 key protocols, i.e., early mobilization, nutritional assistance, and therapeutic (cognitive) activities 3 times daily.	Primary: functional and nutritional status, cognitive function. Secondary: depressive symptoms, cognitive function, and delirium (CAM)	August 2007–April 2009	Gastrointestinal ward	Government, not for-profit.
**Harari 2007[67]**	Before/after study	108 patients admitted for elective orthopedic surgery; age 65+; female 50%	Comprehensive geriatric assessment	Post-operative medical complications, delirium, pressure sores, pain control, delayed mobilization, and inappropriate catheter use.	1. May–July 2003; 2. August 2003–February 2004	Orthopedic ward	Private Not-for profit
**Milisen 2001[68]**	Before/after study	120 patients with a traumatic fracture of proximal femur, median age 81, 80% females.	Education of nursing staff, systematic cognitive screening, consultative services, use of a scheduled pain protocol	Delirium incidence(CAM); severity of delirium; cognitive and functional status (MMSE).	Unclear	Emergency room and 2 traumatological units	Private for profit/Government
**Wong 2005[69]**	Before/after study	99 patients with hip fracture, average age 82 years, female 78%	Ten strategies protocol (oxygen delivery, nutrition and hydration, minimizing medications, regulation of bladder/bowel function, early mobilization, prevention and treatment of major peri- and post-operative complications.	Major outcomes: proportions of subjects with delirium (CAM), discharge destination and length of stay.	15 August and 24 December 2001	Surgical orthopedic setting	Not reported
**Williams 1985[72]**	Before/after study	227 patients, mean age 79 years, female 82%	Preventing approaches related to: strange environment, altered sensory input, loss of control and independence, disruption in life pattern, immobility and pain, and disruption in elimination pattern. Ameliorative approaches related to: mild behaviors suggestive of confusion, sundowning, unsafe behavior, hallucinations or illusions, and fright.	Incidence of delirium or acute confusion identified using a score based on 4 types of behaviors.	unclear	Surgical orthopedic setting	Government, not for-profit.

DSI, Delirium Symptom Interview; CAM, Confusion Assessment Method; MDAS, the Memorial Delirium Assessment Scale; MMS, Mini-Mental State Examination; OBS, Organic Brain Syndrome

The components of the non-pharmacological intervention that were common among the studies are shown in Table 3.

**Table 3:** Elements of the multicomponent non-pharmacological interventions across primary studies.

Study	Staff education	Orientation protocol	Avoidance of sensory deprivation	Multi-disciplinary team	Sleep protocol	Early mobilization	Hydration	Nutrition	Drug list review	Oxygen delivery	Pain control	Elimination of unnecessary medications	Regulation of bowel/ bladder function	Prevention, early detection, and treatment of major postoperative complications	Environmental stimuli	treatment of agitated delirium	Delirium prevention, detection, treatment	Teamwork	Individual care planning	Secondary prevention of falls and fractures	Osteoporosis prophylaxis	Family education	Familiy support	therapetic activities protocol
Lundstrom 2007	X				X	X		X		X	X		X	X			X	X	X	X	X			
Marcantonio 2001						X	X	X		X	X	X	X	X	X	X								
Deschodt 2012				X		X	X	X				X	X	X	X	X								
Björkelund 2010						X	X	X		X	X	X					X							
Milisen 2001	X										X						X	X	X					
Wong 2005		X	X			X	X			X	X	X	X	X		X								
Harari 2007	X					X		X			X		X	X										
Chen 2011						X		X																X
Williams 1985	X	X	X			X					X		X		X		X		X					
Martinez 2012		X	X																			X	X	
Inouye 1999	X	X	X		X	X	X	X																X
Vidan 2009	X	X	X		X	X	X	X	X															
Yoo 2013	X	X	X	X	X	X	X	X	X															
Caplan 2007		X	X				X	X																X
Skrobik 2010	X	X									X					X								
*Cole 1994																		X	X					
*Cole 2002		X	X			X									X			X	X			X	X	
*Pitkala 2006		X				X	X	X	X			X			X				X					X
*Lundstrom 2005	X																	X	X			X		

RCT, randomized controlled trial; CCT, controlled clinical trial; BAS, before-after study;

(*) studies that evaluated non-pharmacological interventions to treat delirium

#### Methodological issues

**Fig 2:**
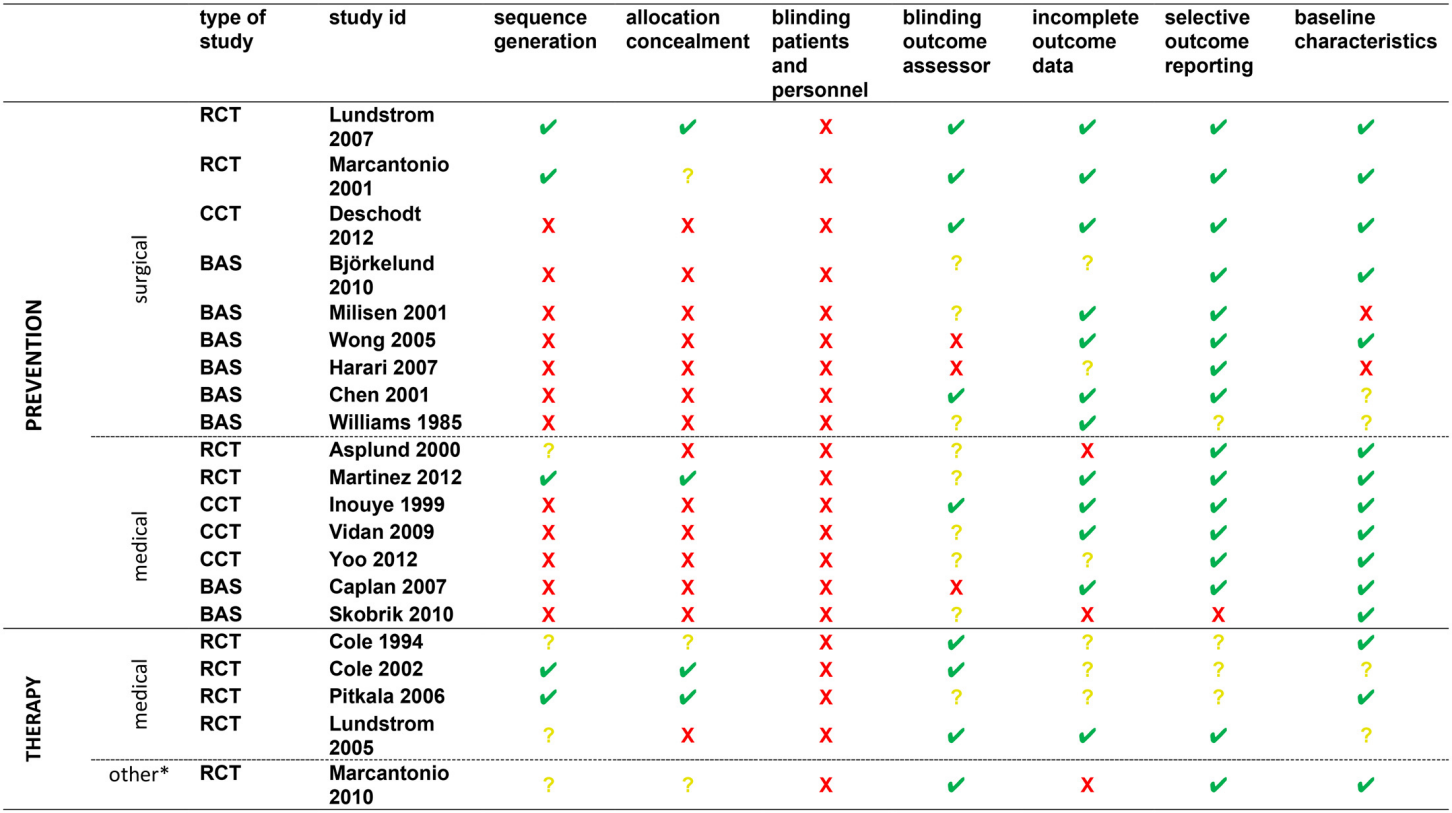
Risk of Bias of Primary Studies of Multicomponent Non-Pharmacological Interventions for Prevention and Treatment of Delirium. ✔low risk of bias? unclear risk of bias **X** high risk of bias; RCT, Randomized Controlled Trial; CCT, Controlled Clinical Trial; BAS before-after studies (*) post-acute skilled nursing facilities.

Given the nature of the intervention, all the comparative studies suffered from performance bias because blinding of patients and personnel could not be carried out. However, all the three trials were immune from detection bias[62–64]. Of the two RCTs, only Lundstrom et al.[62] explicitly reported the method of allocation concealment. The non-randomized studies (CCTs and BA studies) were, by their nature, at risk of selection bias. In two of the four BA studies, the outcome assessor could not be blinded, thus raising the possibility of detection bias. Fig 2 summarizes the risk of bias in each study.

### Efficacy

#### Incidence of delirium

Two RCTs presented data that could be combined, given the similarity between the population samples and the items of the non-pharmacological interventions [62,63]. In fact these two trials had the following interventions in common: comprehensive geriatric assessment, management and rehabilitation, prevention, early detection and treatment of major postoperative complications, oxygen therapy, regulation of bowel/bladder function, nutrition and hydration (Table 3).

The study by Marcantonio et al.[63] reported the cumulative incidence of delirium during hospitalization and used the Delirium Symptom Interview (DSI), the Memorial Delirium Assessment Scale (MDAS), and the Confusion Assessment Method (CAM)[73] to assess delirium. Lundstrom et al.[62]measured delirium based on nurses’ interviews and the modified Organic Brain Syndrome Scale, which was applied at 3–5 days after hospital admission. The pooled results showed that the multicomponent intervention significantly reduced the incidence of delirium by 29% [RR 0.71 (95% CI, 0.59 to 0.86); I^2^0%]. The overall GRADE quality of evidence was judged to be moderate (Table 4).

**Table 4:** GRADE quality of evidence summary table for the comparisons of multicomponent non-pharmacological interventions with usual care for delirium prevention or treatment.

Quality assessment for comparison							No of patients		Effect		Quality	Importance
No of studies	Design	Risk of bias	Inconsistency	Indirectness	Imprecision	Other considerations	Non-pharmacological intervention	Usual care	Relative risk (95% CI)	Absolute		
**Quality assessment for the comparison of non-pharmacological interventions with usual care for the prevention of delirium**												
Delirium incidence in surgical setting												
2[62,63]	RCT	Serious	No serious inconsistency	No serious indirectness	No serious imprecision	None	76/164 (46.3%)	105/161 (65.2%)	0.71 (0.59 to 0.86)	17 fewer per 100 (from 8 fewer to 24 fewer)	+++ Moderate (a)	Critical
Delirium incidence in medical setting (Multicomponent intervention provided by family members)												
1[74]	RCT	Serious	No serious inconsistency	No serious indirectness	Serious	None	8/144 (5.6%)	19/143 (13.3%)	0.42 (0.19 to 0.92)	35 fewer per 100 (from 5 fewer to 48 fewer)	++ Low (b)	Critical
Delirium incidence in medical setting												
1[75]	RCT	Serious	No serious inconsistency	No serious indirectness	Serious	None	6/182 (3.3%)	4/212 (1.9%)	1.75 (0.5 to 6.1)	1 more per 100 (from 1 fewer to 10 more)	+ Very low (c)	Critical
Delirium incidence in medical setting												
2[76,77]	CCT	Serious	No serious inconsistency	No serious indirectness	No serious imprecision	None	62/596 (10.4%)	133/798 (16.7%)	0.65 (0.49 to 0.86)	21 fewer per 100 (from 12 fewer to 30 fewer)	+++ Moderate (d)	Critical
**Quality assessment for the comparison of non-pharmacological interventions with usual care for the treatment of delirium**												
Delirium improvement												
4[78–81]	RCT	Serious	Serious	No serious indirectness	No serious imprecision	None	-	-	Not Pooled	-	+ Very low (e)	Critical
Functional status												
4[78–81]	RCT	Serious	Serious	No serious indirectness	No serious imprecision	None	-	-	Not pooled	-	+ Very low (f)	Critical

^(a)^ Allocation concealment not clear in one study (Lundstrom 2007); both studies exposed to performance bias.

^(b)^ Unclear blinding of outcome assessor and large confidence interval.

^(c)^ Allocation concealment inadequate; unclear blinding of outcome assessor; per-protocol analysis; and large confidence interval.

^(d)^ Not randomized, controlled clinical trials.

^(e)^ Two studies with unclear/inadequate allocation concealment; 3 studies did not report data on delirium improvement, inconsistency of results (1 study in favor of the experimental treatment and 1 with non-significant results).

^(f)^ Two studies with unclear/inadequate allocation concealment; 2 of the 4 studies did not report data on functional status.

**Fig 3:**
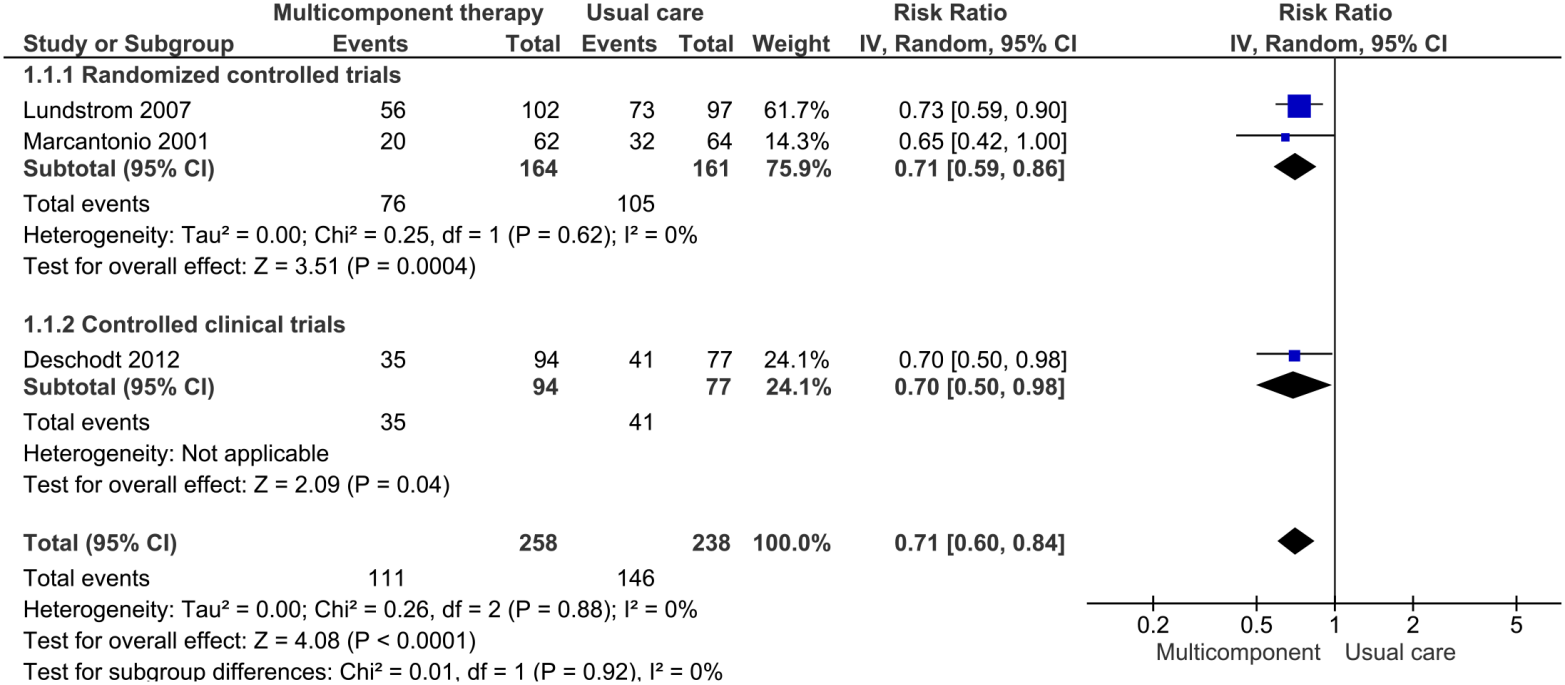
Forest plot of risk ratios comparing multicomponent non-pharmacological interventions vs usual care for delirium prevention in older patients in surgical setting.

Combining the former results with the single CCT[64], which had similar characteristics, yielded pooled results which remained statistically significant with no change in heterogeneity [RR 0.71 (95%CI, 0.60 to 0.84)] (Fig 3).

In three of the 5 BA studies the CAM was employed to assess delirium, while a clinical evaluation was used by Williams et al.[70]and SPMSQ and OBS scale was used by Bjorkelund[65]. Moreover, the assessment time points varied considerably; Wong et al.[69] measured the incidence of delirium every month, Williams et al.[70]measured it within 5 postoperative days, while the remaining studies did not provide the time frame. However, an attempt to pool the data across the studies with patients that received orthopedic surgery, in a meta-analysis, yielded a statistically significant result in favor of the multicomponent interventions [RR 0.57 (95% CI 0.39 to 0.85): I^2^ 27%, P = 0.25][67–70].

#### Evidence of multicomponent, non-pharmacological interventions to prevent delirium in medical setting

Seven studies evaluated the efficacy of multicomponent interventions to prevent delirium in older patients hospitalized in medical departments. Two studies were RCTs [74,75], three were CCTs[76,77,82] and two were BA studies[83,84](Table 5).

**Table 5:** Characteristics of Primary Studies—Non-Pharmacological Interventions for Delirium Prevention in Medical Setting.

Author	Type of study	Population	Intervention	Outcome	Study period	Setting	Funding
**Asplund 2000[75]**	Randomized trial	413 patients; mean age 81 years; 39% men	Acute Geriatric Ward (multidisciplinary team, multicomponent intervention, staff education, early rehabilitation, planning discharge) vs Medical Wards	Primary outcome: delirium incidence (CAM, daily assessment). Secondary outcomes: duration, severity and recurrence of delirium; mortality.	March 18 to December 8,1996	Acute geriatrics ward and general medical wards	Government, private not for profit
**Martinez 2012[74]**	Randomized trial	287 hospitalized patients at intermediate or high risk of delirium (MMSE <24, prior hospitalization, alcoholism or metabolic imbalances on admission); mean age 78; 38% men	Family education, clock and calendar in the room, avoidance of sensory deprivation (glasses, denture and hearing aids), familiar objects in the room, reorientation of patient provided by family members, extended visitation (5 h daily).	Primary outcomes: incidence, severity and duration of delirium during hospitalization (CAM). Secondary outcomes: functional decline, length of stay, mortality, discharge location, need for new social support, number of prescribed drugs	15 September 2009 to 30 May 2010	Internal medicine ward of a Chilean Naval Hospital	Not reported
**Inouye 1999[76]**	Controlled clinical trial	852 subjects at intermediate or high risk of delirium, with at least: visual impairment, severe illness, cognitive impairment, high ratio of blood urea to creatinine); mean age 80; 39% men	Elder Life Program: a trained interdisciplinary team targeted the following risk factors: cognitive impairment, sleep deprivation, immobility, visual impairment, hearing impairment, and dehydration; daily monitoring of adherence.	Primary outcomes: delirium incidence (CAM, every two days) severity, duration, falls, length of stay, cognitive and functional status, cost effectiveness, residential care placement.	March 25, 1995 to March 18, 1998.	General-medicine units at a university-associated hospital	Government
**Vidan 2009[77]**	Controlled clinical trial	542 patients 70 years or older at intermediate or high risk of delirium (at least: visual impairment, acute disease, cognitive impairment, dehydration); mean age 83; 44% men	In the geriatric unit: staff education and specific actions in seven risk areas: orientation, sensory impairment, sleep, mobilization, hydration, nutrition, drug use; daily monitoring of adherence.	Primary outcomes: Incidence of delirium (intensive care delirium screening checklist, assessed 24 h), ICU length of stay, hospital length of stay, antipsychotic use, mortality.	January 15 to December 15, 2007	Geriatric unit and internal medicine ward at University hospital in Madrid, Spain	Private non for profit
**Yoo 2013[82]**	Controlled clinical trial	283 older patients allocated to each group; 43% older than 80 years; male 41%	Interdisciplinary intervention by non-geriatrics specialist physicians vs usual care	Primary outcomes: delirium and transition to a nursing home.	Unclear	Medical setting	Private non for profit
**Caplan 2007[83]**	Before/after study	37 patients at intermediate/high risk of delirium, with at least: MMSE<24, sleep deprivation, any impairment of ADL, vision/hearing impairment, dehydration); mean age: 85; 21% men	The Recruitment of Volunteers to Improve Vitality in the Elderly program: re-orientation; cognitive stimulation; feeding and hydration assistance; vision and hearing protocols.	Primary outcome: delirium incidence (CAM, daily assessment). Secondary outcomes: duration, recurrence and severity of delirium; mortality.	March to August 2003	Geriatric wards at a tertiary referral hospital, Australia.	Government
**Skrobik 2010[84]**	Before/after study	1133 adult patients admitted >24 hours; mean age 63.3 years; 59% men	Teaching staff protocol–based ICU patient assessments targeting non-pharmacological and pharmacological management of pain, sedation and delirium	Primary outcomes: incidence, severity and duration of delirium (CAM, daily assessment +interview of family and nurses and review of medical records of the afternoon/night). Secondary outcomes: functional decline, length of stay, mortality, discharge location, number of prescribed drugs	Before: August 2003 to February 2004; After: April 2005 to November 2005	A single tertiary care adult ICU (Canada)	Government

CAM, Confusion Assessment Method; ICU, Intensive care unit

Yoo et al. evaluated, in a CCT, the efficacy of interdisciplinary intervention by non-geriatricians to prevent delirium. The components of the non-pharmacological intervention in the remaining studies are shown in Table 3. Delirium incidence was a primary outcome in every study except in Asplund et al.[75].

#### Methodological issues

Of the two RCTs, Asplund et al.[75] did not report the exact method of random sequence generation and the personnel could have been aware of the group allocation with block randomization, therefore the study was judged to be at high risk of selection bias. In addition, Asplund et al.[75] was at high risk of attrition bias, because the data were analyzed per protocol.

In the remaining non-randomized studies despite selection bias is a threat to these studies, the baseline characteristics of the population were well balanced. Fig 2 describes the risk of bias table.

### Efficacy

#### Randomized trials

The two RCTs differed substantially in the patient population evaluated. In Asplund et al.[75] where delirium was not a primary outcome and patients were at low risk of developing delirium, non-pharmacological interventions were not able to prevent delirium [RR 1.75 (95% CI 0.50 to 6.10), GRADE quality of evidence was very low]. Conversely, in Martinez et al.[74] patients were at high risk of delirium (age >70 years, a documented cognitive impairment, alcoholism and metabolic imbalances) and the multicomponent intervention, which was performed by family members, was able to reduce the incidence of delirium (evaluated daily with the CAM) by 58% [RR 0.42 (95% CI 0.19 to 0.92)GRADE quality of evidence was low][74].

#### Controlled clinical trials

**Fig 4:**
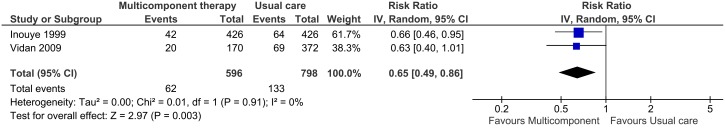
Forest plot of risk ratios comparing multicomponent non-pharmacological interventions vs usual care for delirium prevention in older patients in medical setting.

Two of CCTs investigated very similar patient populations (patients at intermediate/high risk of delirium), and type of multicomponent interventions (Table 3), targeting the same risk factors[76,77]. In both studies, delirium was evaluated daily. The meta-analysis demonstrated a significant risk reduction of 35% of delirium incidence [RR 0.65 (95% CI 0.49 to 0.86); I^**2**^ 0%] (Fig 4). The overall GRADE quality of evidence was judged to be moderate.

The third CCT evaluated an intervention carried out by non-geriatricians which was not effective in preventing delirium[82].

Before-after studies

The two BA studies were different in the patient populations, settings and interventions[84,85] (Table 5) and the interventions in the two studies were not effective in preventing delirium.

#### Evidence of multiple-component, non-pharmacological interventions to treat delirium in hospitalized patients

Four RCTs evaluated the efficacy of non-pharmacological interventions to treat delirium in older patients hospitalized with acute illness in a medical ward trials[78–81] and their characteristics are described in Table 6.

**Table 6:** Characteristics of Included Primary Studies—Non-Pharmacological Interventions for Delirium Treatment.

Study	Type of study	Population	Intervention	Outcome	Study period	Setting	Funding
**Cole 1994[79]**	Randomized trial	88 pts with delirium; mean age 85, male 58%	Consultation by geriatrician or psychiatrist and follow up by a liaison nurse.	Cognitive function (SPMSQ), behavior, mortality rate, length of stay, discharge	8 weeks	Medical ward	Not reported
**Cole 2002[78]**	Randomized trial	227 older patients with delirium admitted to a general medical service; mean age: 82; male 54%.	Consultation by a geriatric internist or psychiatrist and follow up by a liaison nurse. Nursing intervention protocol, Environment, Orientation, Familiarity, Communication, counteraction of immobilization	Primary outcomes: improvement of cognitive status. Secondary outcomes: severity of delirium; length of stay; functional status; death.	8 weeks	Medical ward	Not reported
**Pitkala 2006 [80]**	Randomized trial	174 pts with delirium, age 83, male 25%	Comprehensive geriatric assessment and treatments, Avoiding conventional neuroleptics, Orientation, Physiotherapy, Geriatric interventions (nutrition, hip protection), Comprehensive discharge planning	Primary: mortality or permanent institution, secondary: length of stay, cognitive function, delirium intensity	From September 2001 to November 2002	Medical ward	Not reported
**Lundstrom 2005[81]**	Randomized trial	125 pts with delirium, mean age 81, male 44%.	Multifactorial intervention program (Course in Geriatric Medicine Focusing on Delirium Training Concerning Caregiver-Patient Interaction, Reorganization of Nursing Care, Guidance for Nursing Staff)	Duration of delirium (diagnosis with DSM-IV), mortality, length of stay	8 months	Medical ward	Not reported

DSI, Delirium Symptom Interview; CAM, Confusion Assessment Method; MDAS, the Memorial Delirium Assessment Scale; MMS, Mini-Mental State Examination; SPMSQ, Short Portable Mental Status Questionnaire

The components of the non-pharmacological interventions differed between the studies, with only the individual care planning being a common element to all four studies (Table 3). Three studies[79–81] measured the mortality rate as a primary outcome, while Cole et al.[78] assessed cognitive improvement as a single primary outcome. The length of hospital stay was evaluated as a primary outcome in two studies[79,81].

#### Methodological issues

Two of the RCTs had an adequate method of randomization[78,80]. In the study by Lundstrom et al.[81], the randomization method was unclear, in addition to having a high risk of bias in the allocation concealment (their allocation method depended on the availability of a free bed). The allocation concealment in the study by Cole et al.[79] was unclear. In terms of detection bias three studies displayed low risk of bias and one study [80] had an unclear risk (Fig 2).

### Efficacy

#### Delirium Improvement

The differences in the components of the interventions and the methods used to assess the outcomes precluded a meta-analysis. In the study by Lundstrom et al.[81], delirium was assessed at day 1, 3 and 7 of hospitalization. Complete remission rate at day 7 was significantly higher in patients that received the non-pharmacological intervention [RR 1.58 (95%CI 1.15 to 2.17)]. Pitkala et al.[80] assessed delirium severity using the MDAS scale. Despite the significant statistical difference in favor of the experimental group, the number of incident cases of patients who improved was not reported. Cole et al.[79] used the CAM to assess delirium, but not to determine delirium improvement. In a subsequent publication in 2002[78], the authors assessed the time and rate of improvement of Delirium Index Score, without finding a significant effect of the intervention. The overall GRADE quality of evidence was judged to be very low (Table 4).

#### Functional status

Only two studies evaluated the functional status using the Barthel Index score and results were not statistically significant [78,80].

#### Evidence of multicomponent, non-pharmacological interventions to treat delirium in post-acute care facilities

A cluster RCT evaluated the efficacy of a nurse-led delirium abatement program for post-acute care (PAC) in skilled nursing facilities[86]. The primary outcome was the delirium persistence at 2 weeks and 1 month after post-acute care unit admission.

Methodological limitations of the trial are synthesized in Fig 2.

The intervention allowed a better identification of delirium but was ineffective at reducing delirium[86].

#### Evidence of single-component, non-pharmacological interventions to prevent delirium

Nine studies evaluated the efficacy of single-component non-pharmacological interventions to prevent delirium in acute medical wards (Table 7).

**Table 7:** Characteristics of Included Primary Studies—Single-Component Based Non-Pharmacological Intervention for Delirium Prevention.

Study	Type of study	Population	Intervention	Outcome	Study period	Setting	Funding
**Ono 2011[87]**	Randomized trial	22 patients undergoing esophageal cancer surgery, mean age 63 years, 100% men	Bright light therapy	Physical activity, incidence of post-operative arrhythmia and level of acute delirium (Japanese NEECHAM scale).	February 2006-October 2006	Intensive care unit, post-operative care	Not reported
**Taguchi 2007[88]**	Randomized trial	11 patients undergoing esophageal cancer surgery, mean age 60 years, 100% men	Bright light therapy	Postoperative adjustment of the circadian rhythm, delirium incidence (Japanese NEECHAM scale).	July- December 2003	Intensive care unit, post-operative care	Not reported
**Van Rompaey 2012[89]**	Randomized trial	136 patients in intensive care unit, mean age 59 years, 66% men	Sleep-wake rhythm (ear plugs)	Onset of delirium or confusion (NEECHAM scale), quality of sleep	November 2008-April 2009	Intensive care unit	Not reported
**Lapane 2011[90]**	Cluster randomized trial	3203 residents living in nursing homes, median age 85, 28% men	Geriatric Risk Assessment MedGuide software used to identify resident-specific medications that may contribute to delirium and falls risk.	Incidence of potential delirium, falls, hospitalizations potentially due to adverse drug events, and mortality	2003–2004	Nursing homes	Government
**McCaffrey 2004[91]**	Randomized trial	66 patients undergoing elective hip or knee surgery, mean age 73 years, 100% men	Music therapy	Number of patients with more than one episode of delirium, ambulation readiness profile	7 months	Surgical ward	Not reported
**McCaffrey 2006[92]**	Randomized trial	124 patients undergoing elective hip or knee surgery, mean age 77 years, 18% men	Music therapy	Incidence of delirium, level of patient satisfaction	Not reported	Surgical ward	Not reported
**McCaffrey 2009[93]**	Randomized trial	22 patients undergoing elective hip or knee surgery, mean age 75 years, 36% men	Music therapy	Acute confusion (NEECHAM scale), cognitive function post-surgery (MMSE)	Not reported	Surgical ward	Not reported
**Tabet 2005[94]**	Controlled clinical trial	250 patients, mean age 80 years, 40% men	Staff education	Point prevalence of delirium (modified delirium rating scale). Secondary outcome: recognition and diagnosis of delirium.	December 2001-August 2002	Medical ward	Not reported
**Culp 2003 [95]**	Cluster-randomized trial	98 residents of 7 care homes, mean age 84, 46% men	Hydration management	Incidence of delirium (NEECHAM scale)	-	Nursing-home residence	Not for profit institution
**Colombo 2012 [96]**	Controlled before-after	314 critically-ill patients	Reorientation protocol	Delirium incidence (CAM)	February-June 2008, July-December 2008	Intensive care unit	None

DSI, the Delirium Symptom Interview; CAM, Confusion Assessment Method; NEECHAM, Neelon and Champagne Confusion Scale; MDAS, Memorial Delirium Assessment Scale; MMS, Mini-Mental State Examination

**Fig 5:**
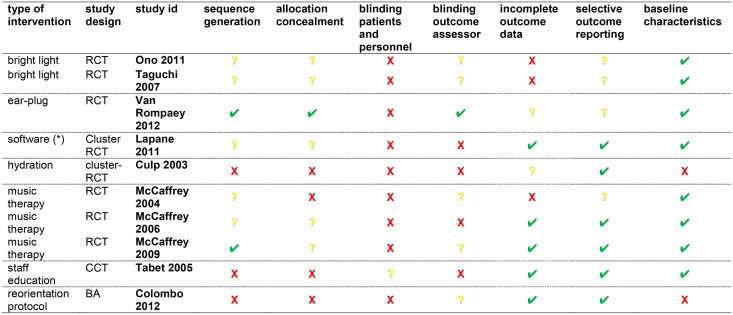
Risk of Bias of Primary Studies of Single Non-Pharmacological Interventions for Prevention of Delirium. ✔low risk of bias? unclear risk of bias **X** high risk of bias; RCT, Randomized Controlled Trial; CCT, Controlled Clinical Trial; BAS before-after studies; (*) Geriatric Risk Assessment MedGuide software.

The risk of bias is summarized in Fig 5.

Two RCTs evaluated the efficacy of Bright Light Therapy in an intensive care unit[87,88]. The intervention reduced the incidence of delirium but without statistically significance[RR 0.29 (95% CI, 0.07 to 1.25)].

Van Rompaey’s randomized trial studied the efficacy of using Ear Plugs to prevent delirium and found a hazard ratio of documenting any benefit for delirium prevention [RR 1.05 (95%CI 0.53 to 2.06)][89].

In a large cluster-RCT Lapane et al.[90] a Geriatric Risk Assessment MedGuide software was used to identify resident-specific medications that may contribute to delirium. The intervention significantly reduced the incidence of delirium in newly admitted residents in the intervention homes than those in usual care homes (hazard ratio 0.42, 95%CI 0.35 to 0.52).

In a cluster RCT, Culp et al.[95] evaluated the efficacy of Hydration management in 98 residents of 7 care homes without documenting any efficacy (RR 0.85, 95% CI 0.18 to 4.00).

McCaffrey et al. studied Music Therapy in three RCTs but results of delirium incidence were not clearly reported[91–93].

In a CCT, Tabet et al.[94]evaluated the efficacy of Staff Education to prevent delirium in a medical ward. The incidence of delirium was significantly lower on the intervention ward despite a wide confidence interval [RR 0.50 (95%CI 0.26 to 0.96)][94].

In a controlled BA study, Colombo et al.[96] evaluated the efficacy of a Reorientation protocol in 314 critically-ill patients admitted to an ICU. Delirium occurrence was significantly lower in the experimental group [RR 0.63 (95%CI 0.44 to 0.91)].

Table 8 displays the summary of finding with GRADE evidence profile for each single component.

**Table 8:** GRADE quality of evidence summary table for the comparisons of single component non-pharmacological interventions with usual care for delirium prevention.

Quality assessment for comparison							No of patients		Effect		Quality	Importance
No of studies	Design	Risk of bias	Inconsistency	Indirectness	Imprecision	Other considerations	Non-pharmacological intervention	Usual care	Relative risk(95% CI)	Absolute		
**Outcome of interest: Delirium incidence**												
**Bright light therapy**												
2[87,88]	RCT	Serious	No serious inconsistency	Serious	Serious	None	2/21(9.5%)	7/20(35%)	0.29 (0.07 to 1.25)	25 fewer per 100 (from 33 fewer to 9 more)	+ Very low (a)	Critical
**Ear plugs**												
1[89]	RCT	Serious	No serious inconsistency	No serious indirectness	Serious	None	14/69(20.3%)	13/67(19.4%)	1.05 (0.53 to 2.06)	1 more per 100 (from 9 fewer to 21 more)	++ Low (b)	Critical
**Geriatric Risk Assessment MedGuide software**												
1[97]	Cluster RCT	Serious	No serious inconsistency	No serious indirectness	No serious indirectness	None	169/1769(9.6%)	264/1552(17%)	HR 0.42 (0.35 to 0.52)	9 fewer per 100 (from 8 fewer to 11 fewer)	+++ Moderate (c)	Critical
**Hydration**												
1[95]	Cluster RCT	Serious	No serious inconsistency	No serious indirectness	Serious	None	3/53(5.7%)	3/45(6.7%)	0.85 (0.18 to 4)	1 fewer per 100 (from 5 fewer to 20 more)	+ Very low (d)	Critical
**Music therapy**												
3[91–93]	RCT	Serious	Serious	No serious indirectness	No serious imprecision	None	-	-	-	-	+ Very low (e)	Critical
**Staff education**												
1[94]	CCT	Serious	No serious inconsistency	No serious indirectness	Serious	None	12/122(9.8%)	25/128(19.5%)	0.5 (0.26 to 0.96)	10 fewer per 100 (from 1 fewer to 14 fewer)	++ Low (f)	Critical
**Reorientation protocol**												
1[96]	BA	Serious	No serious inconsistency	No serious indirectness	Serious		32/144(22.2%)	60/170(35.3%)	0.63 (0.44 to 0.91)	13 fewer per 100 (from 3 fewer to 20 fewer)	+ Very low (g)	Critical

^(a)^ Allocation concealment not clear; blinding of outcome assessor; Japanese with esophageal cancer (indirectness); more than 20% exclusions; large confidence interval.

^(b)^ Unclear number of included patients in final analysis; large confidence interval.

^(c)^ Allocation concealment unclear.

^(d)^ Inadequate randomization; outcome assessor not blinded; unclear final analysis; significant different at baseline (blood urea nitrogen and creatinine ratio).

^(e)^ Inadequate/unclear allocation concealment; outcome assessor not blinded/unclear; results inadequately reported.

^(f)^ High risk of selection bias (controlled clinical trial); outcome assessor not blinded; large confidence interval.

^(g)^ High risk of selection bias (before-after study); outcome assessor not blinded; differences in baseline characteristics (diabetes, sepsis,…); large confidence interval

## Discussion

This systematic review was aimed at identifying systematic reviews and meta-analyses of non-pharmacological interventions used to prevent or treat delirium in patients aged 60 years or older to provide a summary for decision makers and guideline developers.

From 26 SRs or meta-analyses meeting our inclusion criteria, we analyzed data from 31 primary studies published in the last 20 years.

We found evidence of moderate quality supporting the efficacy of multicomponent non-pharmacological interventions to prevent delirium in older patients acutely admitted to a surgical or a medical ward. It must be emphasized, however, that these interventions are effective when administered to patient at intermediate/high-risk of developing delirium. When single component interventions were considered staff-education, reorientation protocol and the Geriatric Risk Assessment MedGuide software were the only interventions that produced significant reduction in delirium prevention albeit a wide confidence interval, likely due to the fact that only one study has been performed for each of them. However, staff-education, reorientation protocol and drug review were present in trials where the multicomponent interventions resulted effective in reducing the delirium incidence.

On the contrary, evidence was insufficient to determine the benefit of multicomponent interventions in the prevention of delirium in other care settings (i.e., nursing homes). Finally, conflicting but mainly negative evidence was found concerning the utility of multicomponent non-pharmacological interventions to *treat* delirium in older medical patients.

### Strengths and weaknesses of the study

This is the first systematic review to gather evidence from different SRs and meta-analyses concerning non-pharmacological treatments for delirium.

Firstly, most of the reviews included in our analysis had different aims and, consequently, the primary studies were distributed among the different reviews requiring the interested reader to consult all of them. For example, the review of Mak et al.[34], focused on prevention of delirium in patients with hip fracture[34]. Skingleyet al.[33] were interested in music as a single component intervention to prevent delirium[33]; Holroyd-Leduc et al.[38]considered both pharmacological and non-pharmacological interventions without examining the single component intervention[38];the review by Hempenius et al.[98] was limited only to delirium prevention[30] (S6 Table shows how the primary studies were distributed among the reviews). Thus, our systematic overview provides a unique tool that synthesizes the evidence on non-pharmacological intervention.

Secondly, unlike previously published reviews, we carefully examined the multicomponent intervention in order to identify the components that were common among different studies and used this information to decide whether it was feasible to perform a meta-analysis. In fact, in the assessment of non-pharmacological interventions for delirium prevention in older surgical patients, we were able to combine 2 RCTs [62,63] together with the recently published CCT [64] and from this combination of studies, we obtained a more precise estimate of the efficacy of the interventions. Similarly, in the setting of acute medical wards, we meta-analyzed Inouye and Vidan’s CCTs[76,77] based on the fact that they had at least seven components in common.

Third, we categorized the studies based on study design, the provision of intervention (for prevention or treatment), and the setting in which the intervention was provided as well as the risk of bias for each study. We believe that this classification approach will facilitate the formulation of clinical questions to assist clinicians to make decisions and to help guideline developers produce recommendations.

Fourth, the strength of evidence is evaluated according to the GRADE items of risk of bias, inconsistency, indirectness, imprecision, and publication bias.

We acknowledge that two studies were recently published that compares to our analysis. The first, was a meta-analysis that considered the efficacy of multicomponent interventions on, among other outcomes, delirium prevention[99]. The study pooled the results of 11 studies including randomized, controlled clinical trials and non-randomized studies and found an OR 0.47 (95% CI, 0.38 to 0.58) in favor of non-pharmacological intervention. The second article is a practice statement from the American Geriatrics Society[100] and it deals with the risk factors, diagnosis, and management of delirium including the use of non-pharmacological intervention for prevention and treatment of postoperative delirium. The authors identified 11 studies including randomized and non-randomized studies and state that the incidence of delirium was reduced but did not perform a meta-analysis. The conclusions of the two paper about multicomponent interventions to prevent delirium are similar to ours although our approach to meta-analyze data considered the setting in which the intervention is provided, the elements that composed the multicomponent interventions and the study design. In addition, we applied the GRADE approach and provide a summary of findings table that can provide the reader a complete account of the confidence of the evidence for the recommendation of multicomponent interventions.

We acknowledge that our review has some limitation. Firstly, the arbitrary age cut-off that may limit the applicability of the evidence from the present overview to patients with less than 60 years of age. Secondly, the lack of assessment of cost-effectiveness reviews does not allow us to reach any conclusions regarding this topic. Thirdly, the studies examined were heterogeneous in terms of intervention, study design, population, outcome and instrument assessment. To address this issue we adopted the best available methodology, i.e. GRADE, to evaluate and synthesize the available evidence.

### Implications for healthcare professionals

Delirium is a geriatric syndrome and as such, it is expected to have a multifactorial etiology in the majority of patients[70,86]. This implies that multicomponent interventions are those with the best chance of being effective. We did find that multicomponent interventions were able to prevent delirium in hospitalized older patients. However, the multicomponent interventions adopted in different studies were quite heterogeneous and therefore difficult to compare. We were able to identify some common elements among different interventions, but no evidence is available to allow either the identification of which multicomponent program is more effective than others, or the relative contribution of each constituent intervention to the positive results in each individual patient. [101].

### Unanswered questions and future research

The process of de-itemizing the multicomponent interventions used to provide the rational to pool meta-analyses may have another implication. For example, in delirium prevention in surgical setting, early mobilization, nutrition and hydration, regulation of bowel/bladder function, and early prevention of complications were the items that randomized studies had in common; in medical setting, in addition to early mobilization, nutrition and hydration, the interventions that the two controlled trials had in common were staff education, orientation protocol, avoidance of sensory deprivation. It is conceivable that these items may have more weight in determining the efficacy. Future studies may consider to design different multicomponent modalities in order to understand which items provide the most important contribution to the efficacy of the multicomponent intervention, how they are interrelated and the resources needed to implement the intervention.

Another point worthy of comment is the fact that the multicomponent interventions work well to *prevent* delirium, but their efficacy to *treat* delirium is at best controversial, with more negative than positive results from the studies evaluated. There is no simple explanation for these findings. One possibility is that while the risk factors for delirium are well characterized[70] and their management can effectively reduce the incidence of new episodes of this geriatric syndrome, the pathophysiology underlying the onset, development and persistence of confusion is not well understood. Several theories have been proposed, including impairment of cerebral metabolism (metabolic encephalopathy), intoxication by drugs, especially those with anticholinergic effects, inflammation, hypercortisolism, and a combination of the former, but no hypothesis has a strong experimental evidence in support of its validity[102].

Therefore, multicomponent interventions investigated in delirium treatment studies are quite similar to those developed for delirium prevention, but they are applied in a very different clinical scenarios. Further research concerning the pathophysiology of delirium is clearly needed to provide the data to support the development of more effective interventions to treat older patients suffering from delirium.

## Conclusions

In older patients at intermediate/high risk of delirium multi-component non-pharmacological interventions as well as some single-components intervention (staff education, reorientation and drug review) reduce the incidence of delirium. Evidence for the role of non-pharmacological interventions in the treatment of delirium is inconclusive.

## Supporting Information

S1 Box List of non-pharmacological interventions to prevent or treat delirium. (DOCX)

S2 Box List of Search strategies. (DOCX)

S1 File Results for secondary outcomes. (DOCX)

S1 Table List of non-pharmacological interventions. (DOCX)

S2 Table Ranking of possible important outcomes when making decisions on delirium prevention. (DOCX)

S3 Table List of excluded reviews with reasons. (DOCX)

S4 Table Methodological quality assessment of the included studies systematic reviews (AMSTAR). (DOCX)

S5 Table Excluded primary studies with reasons. (DOCX)

S6 Table Distribution of Primary Studies (column) across Systematic Reviews (row). (XLSX)
