# Supplementary material for: Efficacy of Non-Pharmacological Interventions to Prevent and Treat Delirium in Older Patients: A Systematic Overview. The SENATOR project ONTOP Series
Source: PLoS One. 2015 Jun 10;10(6):e0123090. doi: 10.1371/journal.pone.0123090 (PMC4465742; doi:10.1371/journal.pone.0123090)
Supplement: S1 Box — (DOCX) [file pone.0123090.s001.docx]

**SI 1 Box. List of non-pharmacological interventions to prevent or treat delirium**

| Education of the staff  Multicomponent interventions  Multidisciplinary team  Early volume repletion  Nutritional intervention  Regulation of bowel function  Regulation of bladder function  Pain treatment  Counteraction of immobilization  Appropriate environmental stimuli  Regular sleep-wake rhythm  Vision protocol  Hearing protocol  Orientation protocol  Family support  Adequate oxygen delivery  Prevention of infection  Prevention and treatment of major postoperative complications  Bright light therapy  Consultative services by specially trained nurses  Surgery as soon as possible  Treatment with PEEP bottle  Daily proactive geriatrics consultation  Music therapy |
| --- |
