# Supplementary material for: Efficacy of Non-Pharmacological Interventions to Prevent and Treat Delirium in Older Patients: A Systematic Overview. The SENATOR project ONTOP Series
Source: PLoS One. 2015 Jun 10;10(6):e0123090. doi: 10.1371/journal.pone.0123090 (PMC4465742; doi:10.1371/journal.pone.0123090)
Supplement: S2 Box — (DOCX) [file pone.0123090.s002.docx]

**SI 2 Box. List of Search strategies**

1. The Cochrane Library

*[Delirium] explode all trees AND delirium*

2. Medline (via Pubmed; ) Montori’s highly specific search strategy was used [BMJ, 2005])

*(MEDLINE[Title/Abstract] OR (systematic[Title/Abstract] AND review[Title/Abstract] OR meta-analysis[Publication Type])) AND (delirium OR (acute confusion) OR ("Delirium"[Mesh]) OR (acute brain syndrome) OR acute organic psychosyndrome OR acute brain syndrome OR metabolic encephalopathy OR clouded state OR clouding of consciousness OR exogenous psychosis OR toxic psychosis OR toxic psychosis)*

3. Embase (via Embase.com)

*('systematic review'/exp OR 'systematic review' OR  'meta analysis'/exp OR 'meta analysis') AND ('delirium'/exp OR delirium OR 'acute confusion'/exp OR 'acute confusion' OR 'cognitive defect'/exp OR 'cognitive defect') AND ('human'/de) AND [embase]/lim*

4. Pshycinfo (via Ovid)

*(exp Delirium/ OR exp Delirium/ or acute confusion.mp. OR acute organic psychosyndrome.mp. OR acute brain syndrome.mp. OR metabolic encephalopathy.mp. OR acute psycho-organic syndrome.mp. OR clouded state.mp. OR clouding of consciousness.mp. OR exogenous psychosis.mp. OR toxic psychosis.mp. OR toxic confusion.mp.) AND (systematic review.mp. OR exp Meta Analysis/ OR MEDLINE.ab.)*

5.CINAHL (via EBSCO)

*(delirium OR acute confusion OR acute organic psychosyndrome OR acute brain syndrome OR metabolic encephalopathy OR acute psycho-organic syndrome OR clouded state OR clouding of consciousness OR exogenous psychosis OR toxic psychosis OR toxic confusion) AND (systematic review OR meta-analysis OR AB medline)*
