# Supplementary material for: Efficacy of Non-Pharmacological Interventions to Prevent and Treat Delirium in Older Patients: A Systematic Overview. The SENATOR project ONTOP Series
Source: PLoS One. 2015 Jun 10;10(6):e0123090. doi: 10.1371/journal.pone.0123090 (PMC4465742; doi:10.1371/journal.pone.0123090)
Supplement: S1 File — (DOCX) [file pone.0123090.s003.docx]

**SI 1 File**

**Results for secondary outcomes (considered important but not critical)**

**1. Evidence of multicomponent, non-pharmacological interventions to prevent delirium in hospitalised surgical older patients**

**Duration and severity of delirium**

In the study by Lundstrom et al., [[1](#_ENREF_1)], the group of patients who received the non-pharmacological intervention showed fewer days with postoperative delirium than patients receiving usual care (5.0±7.1 days vs 10.2±13.3 days, p=0.009).

Marcantonio et al. [[2](#_ENREF_2)] did not report delirium duration data, but concluded that there was no statistical difference between the two groups. In this trial, the median duration of delirium in the control and the intervention groups was 1 day with no statistically significant difference . To determine the severity of the delirium, the authors used the mean Delirium Index score, but no statistical differences were observed during an average of 15 days’ observation.

Milisen et al. [[3](#_ENREF_3)] reported that the duration of delirium was significantly shorter in the intervention cohort (median = 1 day, interquartile range (IQR) = 1) compared with the control cohort (median = 4 days, IQR = 5.5; P = 0.03) and the severity of delirium was significantly less in the control cohort. Wong et al. [[4](#_ENREF_4)] reported that the duration of delirium was not significantly affected by the intervention (median and interquartile range: baseline 5 (2–6) days, post-intervention 3 (2–4) days; P = 0.43); in addition, the study did not assess the severity of delirium. Chen et al. [[5](#_ENREF_5)], Harari et al. [[6](#_ENREF_6)]and Williams et al. [[7](#_ENREF_7)]did not evaluatethedurationandseverityofdelirium.

**Length of hospital stay**

The two RCTs provided contradictory results regarding the hospital length of stay. Lundstrom et al. [[1](#_ENREF_1)] reported that the number of hospital days was lower in the intervention group (MD -0.32 [-0.60 to -0.04]) than in the control group; however, Marcantonio et al. [[2](#_ENREF_2)] found no significant difference between the two groups. The multicomponent intervention had a non-significant reduction of the length of hospital stay, based on the pooled data, with a non-significant heterogeneity (SMD -0.18 [-0.50 to 0.14]; I=50%; p=0.16).

In the BA studies, Harari et al.[[6](#_ENREF_6)] and Wong et al.[[4](#_ENREF_4)] reported a significant reduction of hospital stay in favor of the intervention group, while Chen et al.[[5](#_ENREF_5)], Milisen et al. [[3](#_ENREF_3)]and Williams et al.[[7](#_ENREF_7)]observed no significant difference between the cohorts.

**In-hospital and 1-year mortality**

One RCT [[1](#_ENREF_1)] and three BA studies [[3](#_ENREF_3)[4](#_ENREF_4)[6](#_ENREF_6)] assessed in-hospital mortality but did not find any statistically significant difference.

**2. Evidence of multicomponent, non-pharmacological interventions to prevent delirium in hospitalised patients in medical wards**

**Delirium duration and severity**

Delirium duration, which was measured by Martinez et al. [42] but not by Asplund et al. [41], was not reduced in the intervention group [median (IQR): intervention 2 days (1-2), control 3 days (1-5), P=0.37]. Delirium severity was not evaluated in either of the two randomised trials.

Notwithstanding the similarities between the two CCTs[[8](#_ENREF_8)], the intervention had no consistent effect on severity of delirium and heterogeneity was extremely high (I^2^=98%; p<0.00001).

In the BA studies, the multicomponent intervention reduced delirium severity [mean difference -3.9 (95% CI -7.61, -0.19)], but not delirium duration in Caplan et al. [[9](#_ENREF_9)]). Neither of these outcomes were assessed in Skrobik et al. [[10](#_ENREF_10)].

**Length of hospital stay**

There was no statistically significant difference in this outcome in any of the RCTs. In the BA studies, Skrobik et al. [[10](#_ENREF_10)] found that the intervention reduced the length of stay [mean difference in days -27.9 (95% CI -36.29, -19.51)], whereas Caplan et al. [[9](#_ENREF_9)] did not observe a statistically significant difference between the intervention and control groups.

**In-hospital, 1 and 3 month mortality**

Although mortalitywas measured in all the studies, it was unchanged by the non-pharmacological interventions, except for Skrobik et al. [[10](#_ENREF_10)] who found reduced mortality one-month post-discharge in the treatment group [RR 0.78 (95%CI 0.64, 0.95)],.

**Functional status**

This outcome was evaluated with two different activities of daily living (ADL) assessment scales (Katz Scale and Barthel Index ) and was reported differently in each of the three trials. In their RCT, Asplund et al. [[11](#_ENREF_11)] performed the ADL assessment with the Barthel Index, as per-protocol, three months post-discharge. They observed no statistically significant ADL differences between the two groups. Vidan et al. in their CCT found a 19% decrease in the incidence of functional decline, using the Katz Index [RR 0.81 (95% CI 0.67, 0.97)]. In a BA study, Caplan et al. [REF] detected a non- significant decrease in functional decline from baseline in the intervention group using the Barthel index (SMD -0.60 [-0.06 to 1.27]).

**Cognitive status**

Cognitive status was measured using the MMSE in all three studies, but it was reported differently in each trial. Asplund et al. [[11](#_ENREF_11)] published the median score and interquartile range, at three months, but found no statistically significant difference between groups. Inouye et al. [[12](#_ENREF_12)], in a CCT, expressed the results as the change from baseline (number of patients) in three categories (unchanged, improved by two points and worsened by two points on the MMSE scale). Caplan et al. [[9](#_ENREF_9)], in their BA study, found that the intervention was associated with a positive change in cognitive performance on the MMSE [MD 4.15[(95% CI 1.16, 7.14)].

**3. Evidence of multiple-component, non-pharmacological interventions to treat delirium in hospitalised patients**

**Mortality rate.**

The four RCTs presented mortality rate data. These were not pooled due to differences in the types of interventions. None of the interventions assessed significantly reduced mortality rate except those of Lundstrom et al. [[13](#_ENREF_13)] who reported a statistically significant reduction of mortality rate despite a wide confidence interval [OR 0.19 (95% CI 0.04 to 0.93)].

**Length of hospital stay**

Only three trials provided complete length of hospital stay data [[13-15](#_ENREF_13)]. The treatment in the study by Lundstrom et al. [[13](#_ENREF_13)] led to a significant reduction while in Pitkala et al. [[15](#_ENREF_15)], it was associated with an increase in the length of hospital stay. Cole et al. [[14](#_ENREF_14)] did not observe any significant difference.

**References**

1. Lundstrom M, Olofsson B, Stenvall M, et al. Postoperative delirium in old patients with femoral neck fracture: a randomized intervention study. Aging clinical and experimental research 2007;**19**(3):178-86

2. Marcantonio ER, Flacker JM, Wright RJ, et al. Reducing delirium after hip fracture: a randomized trial. Journal of the American Geriatrics Society 2001;**49**(5):516-22

3. Milisen K, Foreman MD, Abraham IL, et al. A nurse-led interdisciplinary intervention program for delirium in elderly hip-fracture patients. Journal of the American Geriatrics Society 2001;**49**(5):523-32

4. Wong D, Bruce J, GB B. Innovations in Aged Care. Delirium prevention after hip fracture Quality project to prevent delirium after hip fracture. Australasian Journal on Ageing 2005;**24**(3):174 –77

5. Chen CC, Lin MT, Tien YW, et al. Modified hospital elder life program: effects on abdominal surgery patients. Journal of the American College of Surgeons 2011;**213**(2):245-52 doi: 10.1016/j.jamcollsurg.2011.05.004[published Online First: Epub Date]|.

6. Harari D, Hopper A, Dhesi J, et al. Proactive care of older people undergoing surgery ('POPS'): designing, embedding, evaluating and funding a comprehensive geriatric assessment service for older elective surgical patients. Age and ageing 2007;**36**(2):190-6 doi: 10.1093/ageing/afl163[published Online First: Epub Date]|.

7. Inouye SK, Charpentier PA. Precipitating factors for delirium in hospitalized elderly persons. Predictive model and interrelationship with baseline vulnerability. JAMA : the journal of the American Medical Association 1996;**275**(11):852-7

8. Vidan MT, Sanchez E, Alonso M, et al. An intervention integrated into daily clinical practice reduces the incidence of delirium during hospitalization in elderly patients. Journal of the American Geriatrics Society 2009;**57**(11):2029-36 doi: 10.1111/j.1532-5415.2009.02485.x[published Online First: Epub Date]|.

9. Caplan GA, Coconis J, Board N, et al. Does home treatment affect delirium? A randomised controlled trial of rehabilitation of elderly and care at home or usual treatment (The REACH-OUT trial). Age and ageing 2006;**35**(1):53-60 doi: 10.1093/ageing/afi206[published Online First: Epub Date]|.

10. Skrobik Y, Ahern S, Leblanc M, et al. Protocolized intensive care unit management of analgesia, sedation, and delirium improves analgesia and subsyndromal delirium rates. Anesthesia and analgesia 2010;**111**(2):451-63 doi: 10.1213/ANE.0b013e3181d7e1b8[published Online First: Epub Date]|.

11. Asplund K, Gustafson Y, Jacobsson C, et al. Geriatric-based versus general wards for older acute medical patients: a randomized comparison of outcomes and use of resources. Journal of the American Geriatrics Society 2000;**48**(11):1381-8

12. Inouye SK, Bogardus ST, Jr., Charpentier PA, et al. A multicomponent intervention to prevent delirium in hospitalized older patients. The New England journal of medicine 1999;**340**(9):669-76 doi: 10.1056/nejm199903043400901[published Online First: Epub Date]|.

13. Lundstrom M, Edlund A, Karlsson S, et al. A multifactorial intervention program reduces the duration of delirium, length of hospitalization, and mortality in delirious patients. Journal of the American Geriatrics Society 2005;**53**(4):622-8 doi: 10.1111/j.1532-5415.2005.53210.x[published Online First: Epub Date]|.

14. Cole MG, McCusker J, Bellavance F, et al. Systematic detection and multidisciplinary care of delirium in older medical inpatients: a randomized trial. CMAJ : Canadian Medical Association journal = journal de l'Association medicale canadienne 2002;**167**(7):753-9

15. Pitkala KH, Laurila JV, Strandberg TE, et al. Multicomponent geriatric intervention for elderly inpatients with delirium: a randomized, controlled trial. The journals of gerontology. Series A, Biological sciences and medical sciences 2006;**61**(2):176-81
