# Supplementary material for: Efficacy of Non-Pharmacological Interventions to Prevent and Treat Delirium in Older Patients: A Systematic Overview. The SENATOR project ONTOP Series
Source: PLoS One. 2015 Jun 10;10(6):e0123090. doi: 10.1371/journal.pone.0123090 (PMC4465742; doi:10.1371/journal.pone.0123090)
Supplement: S1 Table — (DOCX) [file pone.0123090.s004.docx]

**SI 1 Table. Ranking of possible important outcomes when making decisions on delirium prevention**

| **Answer Options** | **Average result** | **Relative importance** |
| --- | --- | --- |
| incident delirium | **8.3** | **Critical** |
| worsening functional status | **5.7** | **Important** |
| length of hospital stay | **5.4** | **Important** |
| severity of a delirium episode | **4.6** | **Important** |
| cost to health care services | **4.3** | **Important** |
| duration of a delirium episode | **4.2** | **Important** |
| nursing home admission | **4.2** | **Important** |
| quality of life | **4.2** | **Important** |
| use of psychotropic medications | **4.1** | **Important** |
| worsening cognitive status | 3.9 | Not important |
| Death | 3.8 | Not important |
| cost of intervention | 3.6 | Not important |
| incidence of behavioural disturbances | 3.5 | Not important |
| carers psychological morbidity | 2.9 | Not important |
| incidence of dementia | 2.7 | Not important |
| staff psychological morbidity | 2.6 | Not important |
| psychological morbidity | 2.5 | Not important |
