# Supplementary material for: Efficacy of Non-Pharmacological Interventions to Prevent and Treat Delirium in Older Patients: A Systematic Overview. The SENATOR project ONTOP Series
Source: PLoS One. 2015 Jun 10;10(6):e0123090. doi: 10.1371/journal.pone.0123090 (PMC4465742; doi:10.1371/journal.pone.0123090)
Supplement: S2 Table — (DOCX) [file pone.0123090.s005.docx]

**SI 2 Table. Ranking of possible important outcomes when making decisions on delirium therapy**

| **Answer Options** | **Average result** | **Relative importance** |
| --- | --- | --- |
| delirium improvement | **7.5** | **Critical** |
| worsening functional status | **7.6** | **Critical** |
| duration of a delirium episode | **6.4** | **Important** |
| worsening cognitive status | **6.1** | **Important** |
| severity of a delirium episode | **5.9** | **Important** |
| length of hospital stay | **5.7** | **Important** |
| use of psychotropic medications | **5.1** | **Important** |
| quality of life | **5.0** | **Important** |
| Death | **4.6** | **Important** |
| nursing home admission | **4.4** | **Important** |
| incidence of behavioural disturbances | **4.4** | **Important** |
| incident delirium | **4.4** | **Important** |
| carers psychological morbidity | 3.8 | Not important |
| cost to health care services | 3.4 | Not important |
| psychological morbidity | 3.3 | Not important |
| staff psychological morbidity | 3.2 | Not important |
