# Supplementary material for: Efficacy of Non-Pharmacological Interventions to Prevent and Treat Delirium in Older Patients: A Systematic Overview. The SENATOR project ONTOP Series
Source: PLoS One. 2015 Jun 10;10(6):e0123090. doi: 10.1371/journal.pone.0123090 (PMC4465742; doi:10.1371/journal.pone.0123090)
Supplement: S3 Table — (DOCX) [file pone.0123090.s006.docx]

**SI 3 Table. List of excluded reviews with reasons**

| **Study ID** | **Year** | **Reason for exclusion** |
| --- | --- | --- |
| **Azermai^[1](#_ENREF_1)^** | 2012 | Delirium not considered |
| **Ballard**[**^2^**](#_ENREF_2) | 2010 | Delirium not considered |
| **Bartels**[**^3^**](#_ENREF_3) | 2003 | Delirium not considered |
| **Barton**[**^4^**](#_ENREF_4) | 2005 | Delirium not considered |
| **Benoit**[**^5^**](#_ENREF_5) | 2005 | Delirium not considered |
| **Campos**[**^6^**](#_ENREF_6) | 2010 | Review in Portuguese |
| **Chassagne^[7](#_ENREF_7)^** | 2005 | Search strategy not reported |
| **Chevrolet**[**^8^**](#_ENREF_8) | 2007 | Database searched unclear |
| **Clarfield^[9](#_ENREF_9)^** | 2001 | Delirium not considered |
| **Clegg**[**^10^**](#_ENREF_10) | 2011 | Search strategy not reported |
| **Cole**[**^11^**](#_ENREF_11) | 1993 | Non-pharmacological interventions not considered |
| **Corbett**[**^12^**](#_ENREF_12) | 2012 | Delirium not considered |
| **Diana**[**^13^**](#_ENREF_13) | 2003 | Delirium not considered |
| **Eells^[14](#_ENREF_14)^** | 2014 | Music therapy for anxiety prevention (references checked) |
| **Feltrin^[15](#_ENREF_15)^** | 2013 | No systematic search (references checked) |
| **Forbes**[**^16^**](#_ENREF_16) | 2009 | Delirium not considered |
| **Gagnon**[**^17^**](#_ENREF_17) | 2008 | Non-pharmacological interventions not considered |
| **Guenther**[**^18^**](#_ENREF_18) | 2011 | Search strategy not reported |
| **Gustafson**[**^19^**](#_ENREF_19) | 2002 | Review in Swedish |
| **Healey**[**^20^**](#_ENREF_20) | 2012 | Delirium not considered |
| **Hines1**[**^21^**](#_ENREF_21) | 2010 | Delirium not considered |
| **Hines2**[**^22^**](#_ENREF_22) | 2010 | Delirium not considered |
| **Holly**[**^23^**](#_ENREF_23) | 2013 | Overview of reviews (references checked) |
| **Hosie^[24](#_ENREF_24)^** | 2012 | Limited to delirium detection |
| **Keeley^[25](#_ENREF_25)^** | 2009 | Non-pharmacological interventions not considered |
| **Keeley^[26](#_ENREF_26)^** | 2010 | Non-pharmacological interventions not considered |
| **Kong**[**^27^**](#_ENREF_27) | 2009 | Delirium not considered |
| **Köpke^[28](#_ENREF_28)^** | 2010 | This article is not a review. nor it considers delirium |
| **Leentjens^[29](#_ENREF_29)^** | 2005 | Search strategy not reported (reference checked) |
| **Leo**[**^30^**](#_ENREF_30) | 2005 | Non-pharmacological interventions not considered |
| **Locca^[31](#_ENREF_31)^** | 2007 | Non-pharmacological interventions not considered |
| **Markowitz**[**^32^**](#_ENREF_32) | 2008 | Non-pharmacological interventions not considered |
| **McDaniel**[**^33^**](#_ENREF_33) | 2012 | Search strategy not reported (references checked) |
| **Michaud**[**^34^**](#_ENREF_34) | 2004 | Search strategy not reported |
| **Michaud**[**^35^**](#_ENREF_35) | 2007 | Guideline |
| **Mittal**[**^36^**](#_ENREF_36) | 2011 | Search strategy not reported (references checked) |
| **Moyle**[**^37^**](#_ENREF_37) | 2008 | Delirium not considered |
| **Nainar** [**^38^**](#_ENREF_38) | 2011 | Delirium not considered |
| **Nasr** [**^39^**](#_ENREF_39) | 1999 | Not found |
| **Pettersen** [**^40^**](#_ENREF_40) | 2007 | Review in Swedish |
| **Pitkala** [**^41^**](#_ENREF_41) | 2007 | Review of guidelines |
| **Popp** [**^42^**](#_ENREF_42) | 2012 | Search strategy not reported (references checked) |
| **Raijmakers** [**^43^**](#_ENREF_43) | 2011 | Non-pharmacological interventions not considered |
| **Salawu** [**^44^**](#_ENREF_44) | 2009 | No primary studies included |
| **Sanap** [**^45^**](#_ENREF_45) | 2002 | Search strategy not reported |
| **Sieber** [**^46^**](#_ENREF_46) | 2011 | Search strategy not reported |
| **Sockalingam** [**^47^**](#_ENREF_47) | 2005 | No primary studies included |
| **Sumner** [**^48^**](#_ENREF_48) | 1994 | Not found |
| **Teitelbaum** [**^49^**](#_ENREF_49) | 2011 | Non-pharmacological interventions not considered |
| **Theuerkauf** [**^50^**](#_ENREF_50) | 2012 | Search strategy not reported |
| **Van de Glind** [**^51^**](#_ENREF_51) | 2012 | This article is not a review |
| **Van Stijn** [**^52^**](#_ENREF_52) | 2013 | Non-pharmacological interventions not considered |
| **Van Waarde** [**^53^**](#_ENREF_53) | 2004 | Non-pharmacological interventions not considered |
| **Wu** [**^54^**](#_ENREF_54) | 2012 | Non-pharmacological interventions not considered |

**References**

1. Azermai M, Petrovic M, Elseviers MM, et al. Systematic appraisal of dementia guidelines for the management of behavioural and psychological symptoms. Ageing research reviews 2012;**11**(1):78-86.

2. Ballard C, Corbett A. Management of neuropsychiatric symptoms in people with dementia. CNS Drugs 2010;**24**(9):729-39.

3. Bartels SJ, Dums AR, Oxman TE, et al. Evidence-based practices in geriatric mental health care: An overview of systematic reviews and meta-analyses. Psychiatric Clinics of North America 2003;**26**(4):971-90.

4. Barton S, Findlay D, Blake RA. The management of inappropriate vocalisation in dementia: A heirarchical approach. International Journal of Geriatric Psychiatry 2005;**20**(12):1180-86.

5. Benoit M, Brocker P, Clement JP, et al. Behavioral and psychological symptoms in dementia: Description and management. Revue Neurologique 2005;**161**(3):357-66.

6. Campos, Scarpel. Behavioral changes as a nursing care strategy: an integrative review [Portuguese]. Revista Mineira de Enfermagem 2010;**14**(1):110-18.

7. Chassagne P, Druesne L, Bentot C, et al. Mental confusion in the elderly. Presse Medicale 2005;**34**(12):863-68.

8. Chevrolet JC, Jolliet P. Clinical review: agitation and delirium in the critically ill--significance and management. Critical care 2007;**11**(3):214.

9. Clarfield AM. Review: pharmacologic and nonpharmacologic interventions improve outcomes in patients with dementia and for their caregivers. ACP Journal Club 2001;**135**(3):94-94.

10. Clegg A, Young J. In-hospital delirium risk assessment, diagnosis and management; medications to avoid. Italian Journal of Medicine 2011;**5**(2):98-102.

11. Cole MG, Primeau FJ, Francis J. Delirium in hospitalized elderly patients: A meta-analysis. Annals of internal medicine 1993;**119**(SUPPL. 3):87.

12. Corbett A, Smith J, Creese B, et al. Treatment of behavioral and psychological symptoms of Alzheimer's disease. Current Treatment Options in Neurology 2012;**14**(2):113-25.

13. Diana G. Clinical trials for behavioral and psychological symptoms of dementia (BPSD): Methodological and regulatory issues. Annali dell'Istituto Superiore di Sanita 2003;**39**(2):267-74.

14. Lazarus Hr Fau - Hagens JH, Hagens JH. Prevention of psychosis following open-heart surgery. (0002-953X (Print)).

15. Leslie DL, Zhang Y Fau - Bogardus ST, Bogardus St Fau - Holford TR, et al. Consequences of preventing delirium in hospitalized older adults on nursing home costs. (0002-8614 (Print)).

16. Forbes D, Culum I, Lischka AR, et al. Light therapy for managing cognitive, sleep, functional, behavioural, or psychiatric disturbances in dementia. Cochrane Database of Systematic Reviews 2009(4).

17. Gagnon PR. Treatment of delirium in supportive and palliative care. Current opinion in supportive and palliative care 2008;**2**(1):60-6.

18. Guenther U, Radtke FM. Delirium in the postanaesthesia period. Current Opinion in Anaesthesiology 2011;**24**(6):670-75.

19. Gustafson Y, Lundstrom M, Bucht G, et al. [Delirium in old age can be prevented and treated]. Tidsskrift for den Norske laegeforening : tidsskrift for praktisk medicin, ny raekke 2002;**122**(8):810-4.

20. Healey F, Darowski A. Older patients and falls in hospital. Clinical Risk 2012;**18**(5):170-76.

21. Hines S, McCrow J, Abbey J, et al. Thickened fluids for people with dementia in residential aged care facilities. International Journal of Evidence-Based Healthcare 2010;**8**(4):252-55.

22. Hines S, Wilson J, McCrow J, et al. Oral liquid nutritional supplements for people with dementia in residential aged care facilities. International Journal of Evidence-Based Healthcare 2010;**8**(4):248-51.

23. Lundstrom M, Edlund A Fau - Lundstrom G, Lundstrom G Fau - Gustafson Y, et al. Reorganization of nursing and medical care to reduce the incidence of postoperative delirium and improve rehabilitation outcome in elderly patients treated for femoral neck fractures. (0283-9318 (Print)).

24. McCusker J, Cole M Fau - Dendukuri N, Dendukuri N Fau - Belzile E, et al. Delirium in older medical inpatients and subsequent cognitive and functional status: a prospective study. (0820-3946 (Print)).

25. Keeley PW. Delirium at the end of life. Clinical evidence 2009;**2009**.

26. Keeley P. Delirium at the end of life. American Family Physician 2010;**81**(10):1260-61.

27. Kong TK. Safe and effective use of digoxin in old age. CME Journal Geriatric Medicine 2010;**12**(2):86-97.

28. Köpke S, Richter T, Filz M, et al. Psychosocial interventions to reduce antipsychotic drugs in care home residents: systematic review... Fourth European Nursing Congress. Journal of Clinical Nursing 2010;**19**:23-23.

29. Leentjens AF, van der Mast RC. Delirium in elderly people: an update. Current opinion in psychiatry 2005;**18**(3):325-30.

30. Leo RJ, Baer D. Delirium associated with baclofen withdrawal: A review of common presentations and management strategies. Psychosomatics 2005;**46**(6):503-07.

31. Locca JF, Zumbach S, Bula C, et al. [Management of elderly patients with delirium or dementia in Swiss nursing homes]. Revue medicale suisse 2007;**3**(132):2519-20, 22, 24 passim.

32. Markowitz JD, Narasimhan M. Delirium and antipsychotics: a systematic review of epidemiology and somatic treatment options. Psychiatry (Edgmont (Pa : Township)) 2008;**5**(10):29-36.

33. McDaniel M, Brudney C. Postoperative delirium: Etiology and management. Current opinion in critical care 2012;**18**(4):372-76.

34. Michaud L, Burnand B, Stiefel F. Taking care of the terminally ill cancer patient: Delirium as a symptom of terminal disease. Annals of Oncology 2004;**15**(SUPPL. 4):iv199-iv203.

35. Michaud L, Bula C, Berney A, et al. Delirium: Guidelines for general hospitals. Journal of Psychosomatic Research 2007;**62**(3):371-83.

36. Mittal V, Muralee S, Williamson D, et al. Delirium in the elderly: A comprehensive review. American Journal of Alzheimer's Disease and other Dementias 2011;**26**(2):97-109.

37. Moyle W, Olorenshaw R, Wallis M, et al. Best practice for the management of older people with dementia in the acute care setting: a review of the literature. International Journal of Older People Nursing 2008;**3**(2):121-30.

38. Nainar PG, Green DW. Post-operative cognitive dysfunction & the older patient. CME Journal Geriatric Medicine 2011;**13**(2):49-53.

39. Nasr SZ, Osterweil D. The nonpharmacologic management of agitation in the nursing home: a consensus approach. Annals of Long Term Care 1999;**7**(5):171-80.

40. Pettersen R, Wyller TB. [Rehabilitation integrated in acute medical treatment]. Tidsskrift for den Norske laegeforening : tidsskrift for praktisk medicin, ny raekke 2007;**127**(5):600-3.

41. Pitkala KH, Laurila JV. Managing delirium in hospitalized elderly patients. Future Neurology 2007;**2**(3):317-27.

42. Popp J, Arlt S. Prevention and treatment options for postoperative delirium in the elderly. Current opinion in psychiatry 2012;**25**(6):515-21.

43. Raijmakers NJ, van Zuylen L, Costantini M, et al. Artificial nutrition and hydration in the last week of life in cancer patients. A systematic literature review of practices and effects. Annals of oncology : official journal of the European Society for Medical Oncology / ESMO 2011;**22**(7):1478-86.

44. Salawu FK, Danburam A, Ogualili P. Delirium in learning disability: Case series and literature review. Annals of African medicine 2009;**8**(3):139-46.

45. Sanap MN, Worthley LI. Neurologic complications of critical illness: part I. Altered states of consciousness and metabolic encephalopathies. Critical care and resuscitation : journal of the Australasian Academy of Critical Care Medicine 2002;**4**(2):119-32.

46. Sieber FE, Barnett SR. Preventing Postoperative Complications in the Elderly. Anesthesiology Clinics 2011;**29**(1):83-97.

47. Sockalingam S, Parekh N, Bogoch, II, et al. Delirium in the postoperative cardiac patient: a review. Journal of cardiac surgery 2005;**20**(6):560-7.

48. Sumner AD, Simons RJ. Delirium in the hospitalized elderly. Cleveland Clinic journal of medicine 1994;**61**(4):258-62.

49. Teitelbaum J, Ayoub O, Skrobik Y. A critical appraisal of sedation, analgesia and delirium in neurocritical care. Canadian Journal of Neurological Sciences 2011;**38**(6):815-25.

50. Theuerkauf N, Guenther U, Putensen C. Postoperative delirium in the PACU and intensive care unit. Trends in Anaesthesia and Critical Care 2012;**2**(4):148-55.

51. Van De Glind E, Stott D, Hooft L, et al. Evidence-based medicine in geriatrics. European Geriatric Medicine 2012;**3**:S14-S15.

52. Van Stijn MFM, Korkic-Halilovic I, Bakker MSM, et al. Preoperative nutrition status and postoperative outcome in elderly general surgery patients: A systematic review. Journal of Parenteral and Enteral Nutrition 2013;**37**(1):37-43.

53. van Waarde JA, van der Mast RC. Delirium in learning disability: Case series and literature review. British Journal of Learning Disabilities 2004;**32**(3):123-27.

54. Wu CH, Chang CI, Chen CY. Overview of studies related to geriatric syndrome in Taiwan. Journal of Clinical Gerontology and Geriatrics 2012;**3**(1):14-20.
