# Supplementary material for: Efficacy of Non-Pharmacological Interventions to Prevent and Treat Delirium in Older Patients: A Systematic Overview. The SENATOR project ONTOP Series
Source: PLoS One. 2015 Jun 10;10(6):e0123090. doi: 10.1371/journal.pone.0123090 (PMC4465742; doi:10.1371/journal.pone.0123090)
Supplement: S4 Table — (DOCX) [file pone.0123090.s007.docx]

**SI 4 Table. Methodological quality assessment of the included studies systematic reviews (AMSTAR)**

|  |  | **AMSTAR Items** | | | | | | | | | | | |
| --- | --- | --- | --- | --- | --- | --- | --- | --- | --- | --- | --- | --- | --- |
|  |  | **1** | **2** | **3** | **4** | **5** | **6** | **7** | **8** | **9** | **10** | **11** | **Rating** |
| Alway 2013 | | No | No | No | No | No | Yes | No | No | No | No | No | 1 |
| Bitsch 2004 | | No | No | No | No | No | Yes | No | No | No | No | No | 1 |
| Carr 2013 | | No | No | Yes | No | No | Yes | No | No | No | No | No | 2 |
| Clegg 2014 | | Yes | Yes | Yes | Yes | Yes | Yes | Yes | Yes | Yes | No | Yes | 10 |
| Cole 1996 | | No | Yes | Yes | No | No | Yes | Yes | No | No | No | No | 4 |
| Cole 1998 | | No | Yes | Yes | No | No | Yes | Yes | No | No | No | No | 4 |
| Cole 1999 | | No | Yes | Yes | No | No | Yes | Yes | No | No | No | No | 4 |
| Conn 2001 | | No | Yes | No | No | No | Yes | No | No | No | No | No | 2 |
| Fick 2002 | | No | No | No | No | No | Yes | Yes | No | No | No | No | 2 |
| Fox 2012 | | No | Yes | Yes | No | No | Yes | Yes | Yes | Yes | No | No | 6 |
| Gonzales 2003 | | No | Yes | No | No | No | Yes | No | No | No | No | No | 2 |
| Greer 2011 | | No | Yes | Yes | No | No | Yes | Yes | Yes | Yes | No | Yes | 7 |
| Grigoryan 2014 | | No | Yes | Yes | No | No | Yes | Yes | Yes | Yes | Yes | No | 7 |
| Hempenius 2011 | | No | Yes | Yes | No | No | Yes | Yes | Yes | Yes | Yes | No | 7 |
| Holroyd-Leduc 2010 | | No | No | Yes | No | No | Yes | No | No | No | No | No | 2 |
| Inouye 2014 | | No | Yes | Yes | No | No | Yes | Yes | No | No | No | No | 4 |
| Mak 2010 | | No | Yes | Yes | No | No | Yes | Yes | No | No | No | No | 4 |
| Marik 2006 | | No | No | No | No | No | Yes | No | No | No | No | No | 1 |
| Milisen 2005 | | No | No | Yes | No | No | Yes | Yes | No | No | No | No | 3 |
| Morrison 1998 | | No | Yes | No | No | No | Yes | No | No | No | No | No | 2 |
| Moyce 2014 | | No | Yes | No | No | No | Yes | Yes | No | Yes | Yes | No | 5 |
| Reston 2012 | | No | Yes | Yes | No | No | Yes | Yes | No | No | No | No | 4 |
| Siddiqi 2007 | | Yes | Yes | Yes | Yes | Yes | Yes | Yes | Yes | Yes | Yes | Yes | 11 |
| Skingley 2010 | | No | Yes | No | No | No | Yes | No | No | No | No | No | 2 |
| Weber 2004 | | No | Yes | Yes | No | No | Yes | Yes | No | No | No | No | 4 |
| Zhang 2013 | | No | Yes | Yes | Yes | No | Yes | Yes | Yes | Yes | Yes | Yes | 9 |

AMSTAR comprises the following items:

1. ‘a priori’ design provided;

2. duplicate study selection/data extraction;

3. comprehensive literature search;

4. status of publication as inclusion criteria (i.e., grey or unpublished literature);

5. list of studies included/excluded provided;

6. characteristics of included studies documented;

7. scientific quality assessed and documented;

8. appropriate formulation of conclusions (based on methodological rigor and scientific quality of the studies);

9. appropriate methods of combining studies (homogeneity test, effect model used and sensitivity analysis);

10. assessment of publication bias (graphic and/or statistical test); and

11. conflict of interest statement.

Items were scored as “Yes”, “No”, “Can’t Answer” or “Not Applicable”.
