# Supplementary material for: Efficacy of Non-Pharmacological Interventions to Prevent and Treat Delirium in Older Patients: A Systematic Overview. The SENATOR project ONTOP Series
Source: PLoS One. 2015 Jun 10;10(6):e0123090. doi: 10.1371/journal.pone.0123090 (PMC4465742; doi:10.1371/journal.pone.0123090)
Supplement: S5 Table — (DOCX) [file pone.0123090.s008.docx]

**SI 5 Table. Excluded primary studies with reasons**

| **Study** | **Year** | **Reason for exclusion** |
| --- | --- | --- |
| **Aakerlund** [**^1^**](#_ENREF_1) | 1994 | Not a comparative study. |
| **Aizawa** [**^2^**](#_ENREF_2) | 2002 | This study used a pharmacological treatment as an intervention. |
| **Akunne** [**^3^**](#_ENREF_3) | 2012 | An economic analysis of primary studies. |
| **Bogardus** [**^4^**](#_ENREF_4) | 2003 | This was a six-month follow-up study of Inouye 1999. |
| **Budd** [**^5^**](#_ENREF_5) | 1974 | Patients ranged between 23 and 63 years of age. Delirium data were not reported. |
| **Caplan** [**^6^**](#_ENREF_6) | 2006 | A study of home versus hospital care. |
| **Cole** [**^7^**](#_ENREF_7) | 1991 | Delirium incidence was not an outcome. |
| **Egbert** [**^8^**](#_ENREF_8) | 1990 | This study used a pharmacological treatment as an intervention. |
| **Fick** [**^9^**](#_ENREF_9) | 2000 | This was an observational study without any intervention. |
| **Flaherty** [**^10^**](#_ENREF_10) | 2011 | A comment on a retrospective observational study. |
| **Gagnon** [**^11^**](#_ENREF_11) | 2012 | Multicomponent non-pharmacological interventions used as a palliative care for terminally cancer patients |
| **Girard** [**^12^**](#_ENREF_12) | 2008 | The type of intervention was paired sedation and ventilator weaning protocol for mechanically ventilated patients in intensive care. |
| **González-Montalvo** [**^13^**](#_ENREF_13) | 2010 | Delirium not considered. |
| **Gustafson** [**^14^**](#_ENREF_14) | 1991 | This study used a historical control. |
| **Inouye** [**^15^**](#_ENREF_15) | 1993 | Delirium incidence was not an outcome of interest. |
| **Inouye** [**^16^**](#_ENREF_16) | 2003 | This was a follow-up study of Inouye 1999. |
| **Kamdar** [**^17^**](#_ENREF_17) | 2013 | The median age was 54 with interquartile range 43-63. |
| **Koizumi** [**^18^**](#_ENREF_18) | 1988 | Study with serious methodological flaws: subjects selected without a specific inclusion criteria; unclear study design; unclear allocation to treatment. |
| **Kolanowski** [**^19^**](#_ENREF_19) | 2011 | This was a study protocol. |
| **Landefeld** [**^20^**](#_ENREF_20) | 1995 | Delirium incidence was not an outcome. |
| **Layne** [**^21^**](#_ENREF_21) | 1971 | No non-pharmacological treatment considered. |
| **Lazarus** [**^22^**](#_ENREF_22) | 1968 | This was an observational study without any interventions. |
| **Leslie** [**^23^**](#_ENREF_23) | 2005 | This was an economic analysis of Inouye 1999. |
| **Lundstrom** [**^24^**](#_ENREF_24) | 1999 | This study used an historical control. |
| **McCusker** [**^25^**](#_ENREF_25) | 2001 | This was an observational study without any interventions. |
| **Meagher** [**^26^**](#_ENREF_26) | 1996 | The mean age of patients was less than 65 years. There was no control group and the number of non-pharmacological interventions differed between the two delirium groups. |
| **Mehta** [**^27^**](#_ENREF_27) | 2013 | Experimental treatment was mainly based on pharmacologic treatment. |
| **Mentes** [**^28^**](#_ENREF_28) | 2003 | Initial analysis of an included study (Culp 2003) |
| **Milisen** [**^29^**](#_ENREF_29) | 1998 | Delirium incidence was not an outcome. |
| **Milisen** [**^30^**](#_ENREF_30) | 2005 | This was a systematic review. |
| **Mouchoux** [**^31^**](#_ENREF_31) | 2011 | This was a study protocol. |
| **Nagley** [**^32^**](#_ENREF_32) | 1986 | The SPMSQ questionnaire was used to screen for delirium during patient recruitment. |
| **Naughton** [**^33^**](#_ENREF_33) | 2005 | Data were reported at 4 and 9 months post-discharge. but not during hospitalization. |
| **Needham** [**^34^**](#_ENREF_34) | 2010 | Before-after study with a historical control. |
| **Rizzo** [**^35^**](#_ENREF_35) | 2001 | This was an economic analysis of Inouye 1999. |
| **Robinson** [**^36^**](#_ENREF_36) | 2008 | This study used an historical control. |
| **Rockwood** [**^37^**](#_ENREF_37) | 1994 | This was a diagnostic accuracy study. |
| **Rubin** [**^38^**](#_ENREF_38) | 2011 | This was a before-after study using historical controls |
| **Schindler** [**^39^**](#_ENREF_39) | 1989 | The mean age of patients was less than 65 years. |
| **Schweickert** [**^40^**](#_ENREF_40) | 2009 | Patients’ mean age < 60 years; type of intervention was daily interruption of sedation combined with physical and occupational therapy for mechanically ventilated patients. |
| **Siepe** [**^41^**](#_ENREF_41) | 2011 | The experimental group was mainly based on pharmacologic treatment |
| **Stenvall** [**^42^**](#_ENREF_42) | 2007 | This was a follow-up study of Lundstrom 2007. |
| **Stenvall** [**^43^**](#_ENREF_43) | 2012 | This study used subgroup analyses. |
| **Ushida** [**^44^**](#_ENREF_44) | 2009 | This study used a pharmacological treatment as an intervention. |
| **Vidan** [**^45^**](#_ENREF_45) | 2005 | Delirium incidence was not reported. |
| **Voller** [**^46^**](#_ENREF_46) | 2007 | Apparently a before after study. However, the information describing the study design and the baseline population characteristics was extremely limited. In addition. the period of recruitment was unknown and it was unclear if the controls were historical. |
| **Wanich** [**^47^**](#_ENREF_47) | 1992 | The patient population was a mix of subjects with delirium and subjects at risk of developing delirium. |

**References**

1. Aakerlund LP, Rosenberg J. Postoperative delirium: treatment with supplementary oxygen. (0007-0912 (Print)).

2. Aizawa K, Kanai T Fau - Saikawa Y, Saikawa Y Fau - Takabayashi T, et al. A novel approach to the prevention of postoperative delirium in the elderly after gastrointestinal surgery. (0941-1291 (Print)).

3. Akunne A, Murthy L, Young J. Cost-effectiveness of multi-component interventions to prevent delirium in older people admitted to medical wards. Age and ageing 2012;**41**(3):285-91.

4. Bogardus ST, Desai MM, Williams CS, et al. The effects of a targeted multicomponent delirium intervention on postdischarge outcomes for hospitalized older adults. The American journal of medicine 2003;**114**(5):383-90.

5. Budd S Fau - Brown W, Brown W. Effect of a reorientation technique on postcardiotomy delirium. (0029-6562 (Print)).

6. Caplan GA, Coconis J, Board N, et al. Does home treatment affect delirium? A randomised controlled trial of rehabilitation of elderly and care at home or usual treatment (The REACH-OUT trial). Age and ageing 2006;**35**(1):53-60.

7. Cole MG, Fenton Fr Fau - Engelsmann F, Engelsmann F Fau - Mansouri I, et al. Effectiveness of geriatric psychiatry consultation in an acute care hospital: a randomized clinical trial. (0002-8614 (Print)).

8. Egbert AM, Parks Lh Fau - Short LM, Short Lm Fau - Burnett ML, et al. Randomized trial of postoperative patient-controlled analgesia vs intramuscular narcotics in frail elderly men. (0003-9926 (Print)).

9. Fick D, Foreman M. Consequences of not recognizing delirium superimposed on dementia in hospitalized elderly individuals. (0098-9134 (Print)).

10. Flaherty JH, Little MO. Matching the environment to patients with delirium: lessons learned from the delirium room, a restraint-free environment for older hospitalized adults with delirium. (1532-5415 (Electronic)).

11. Gagnon P, Allard P, Gagnon B, et al. Delirium prevention in terminal cancer: assessment of a multicomponent intervention. Psycho-oncology 2012;**21**(2):187-94.

12. Girard TD, Kress JP, Fuchs BD, et al. Efficacy and safety of a paired sedation and ventilator weaning protocol for mechanically ventilated patients in intensive care (Awakening and Breathing Controlled trial): a randomised controlled trial. The Lancet 2008;**371**(9607):126-34.

13. Gonzalez-Montalvo JI, Alarcon T, Mauleon JL, et al. The orthogeriatric unit for acute patients: a new model of care that improves efficiency in the management of patients with hip fracture. Hip international : the journal of clinical and experimental research on hip pathology and therapy 2010;**20**(2):229-35.

14. Gustafson Y, Brannstrom B Fau - Berggren D, Berggren D Fau - Ragnarsson JI, et al. A geriatric-anesthesiologic program to reduce acute confusional states in elderly patients treated for femoral neck fractures. (0002-8614 (Print)).

15. Inouye SK, Wagner Dr Fau - Acampora D, Acampora D Fau - Horwitz RI, et al. A controlled trial of a nursing-centered intervention in hospitalized elderly medical patients: the Yale Geriatric Care Program. (0002-8614 (Print)).

16. Inouye SK, Bogardus St Jr Fau - Williams CS, Williams Cs Fau - Leo-Summers L, et al. The role of adherence on the effectiveness of nonpharmacologic interventions: evidence from the delirium prevention trial. (0003-9926 (Print)).

17. Kamdar BB, King Lm Fau - Collop NA, Collop Na Fau - Sakamuri S, et al. The effect of a quality improvement intervention on perceived sleep quality and cognition in a medical ICU. (1530-0293 (Electronic)).

18. Koizumi J, Shiraishi H Fau - Ofuku K, Ofuku K Fau - Suzuki T, et al. Duration of delirium shortened by the correction of electrolyte imbalance. (0912-2036 (Print)).

19. Kolanowski AM, Fick DM, Litaker MS, et al. Study protocol for the recreational stimulation for elders as a vehicle to resolve delirium superimposed on dementia (Reserve For DSD) trial. Trials 2011;**12**:119.

20. Landefeld CS, Palmer Rm Fau - Kresevic DM, Kresevic Dm Fau - Fortinsky RH, et al. A randomized trial of care in a hospital medical unit especially designed to improve the functional outcomes of acutely ill older patients. (0028-4793 (Print)).

21. Layne Ol Jr Fau - Yudofsky SC, Yudofsky SC. Postoperative psychosis in cardiotomy patients. The role of organic and psychiatric factors. (0028-4793 (Print)).

22. Lazarus Hr Fau - Hagens JH, Hagens JH. Prevention of psychosis following open-heart surgery. (0002-953X (Print)).

23. Leslie DL, Zhang Y Fau - Bogardus ST, Bogardus St Fau - Holford TR, et al. Consequences of preventing delirium in hospitalized older adults on nursing home costs. (0002-8614 (Print)).

24. Lundstrom M, Edlund A Fau - Lundstrom G, Lundstrom G Fau - Gustafson Y, et al. Reorganization of nursing and medical care to reduce the incidence of postoperative delirium and improve rehabilitation outcome in elderly patients treated for femoral neck fractures. (0283-9318 (Print)).

25. McCusker J, Cole M Fau - Dendukuri N, Dendukuri N Fau - Belzile E, et al. Delirium in older medical inpatients and subsequent cognitive and functional status: a prospective study. (0820-3946 (Print)).

26. Meagher DJ, O'Hanlon D, O'Mahony E, et al. The use of environmental strategies and psychotropic medication in the management of delirium. The British Journal of Psychiatry 1996;**168**(4):512-15.

27. Mehta S, Burry L Fau - Cook D, Cook D Fau - Fergusson D, et al. Daily sedation interruption in mechanically ventilated critically ill patients cared for with a sedation protocol: a randomized controlled trial. (1538-3598 (Electronic)).

28. Mentes JC, Culp K. Reducing hydration-linked events in nursing home residents. (1054-7738 (Print)).

29. Milisen K, Abraham Il Fau - Broos PL, Broos PL. Postoperative variation in neurocognitive and functional status in elderly hip fracture patients. (0309-2402 (Print)).

30. Milisen K, Lemiengre J Fau - Braes T, Braes T Fau - Foreman MD, et al. Multicomponent intervention strategies for managing delirium in hospitalized older people: systematic review. (0309-2402 (Print)).

31. Mouchoux C, Rippert P, Duclos A, et al. Impact of a multifaceted program to prevent postoperative delirium in the elderly: the CONFUCIUS stepped wedge protocol. BMC geriatrics 2011;**11**:25.

32. Nagley SJ. Predicting and preventing confusion in your patients. (0098-9134 (Print)).

33. Naughton BJ, Saltzman S Fau - Ramadan F, Ramadan F Fau - Chadha N, et al. A multifactorial intervention to reduce prevalence of delirium and shorten hospital length of stay. (0002-8614 (Print)).

34. Needham DM, Korupolu R, Zanni JM, et al. Early physical medicine and rehabilitation for patients with acute respiratory failure: a quality improvement project. Archives of physical medicine and rehabilitation 2010;**91**(4):536-42.

35. Rizzo JA, Bogardus St Jr Fau - Leo-Summers L, Leo-Summers L Fau - Williams CS, et al. Multicomponent targeted intervention to prevent delirium in hospitalized older patients: what is the economic value? (0025-7079 (Print)).

36. Robinson S, Rich C, Weitzel T, et al. Delirium Prevention for Cognitive, Sensory, and Mobility Impairments. Research and Theory for Nursing Practice 2008;**22**(2):103-13.

37. Rockwood K, Cosway S Fau - Stolee P, Stolee P Fau - Kydd D, et al. Increasing the recognition of delirium in elderly patients. (0002-8614 (Print)).

38. Rubin FH, Neal K, Fenlon K, et al. Sustainability and scalability of the hospital elder life program at a community hospital. Journal of the American Geriatrics Society 2011;**59**(2):359-65.

39. Schindler BA, Shook J Fau - Schwartz GM, Schwartz GM. Beneficial effects of psychiatric intervention on recovery after coronary artery bypass graft surgery. (0163-8343 (Print)).

40. Schweickert. Early physical and occupational therapy in mechanically ventilated, critically ill patients: a randomised controlled trial. 2009.

41. Siepe M, Pfeiffer T, Gieringer A, et al. Increased systemic perfusion pressure during cardiopulmonary bypass is associated with less early postoperative cognitive dysfunction and delirium. European Journal of Cardio-Thoracic Surgery 2011;**40**(1):200-07.

42. Stenvall M, Olofsson B, Nyberg L, et al. Improved performance in activities of daily living and mobility after a multidisciplinary postoperative rehabilitation in older people with femoral neck fracture: a randomized controlled trial with 1-year follow-up. Journal of rehabilitation medicine : official journal of the UEMS European Board of Physical and Rehabilitation Medicine 2007;**39**(3):232-8.

43. Stenvall M, Berggren M, Lundstrom M, et al. A multidisciplinary intervention program improved the outcome after hip fracture for people with dementia--subgroup analyses of a randomized controlled trial. Archives of gerontology and geriatrics 2012;**54**(3):e284-9.

44. Ushida T, Yokoyama T Fau - Kishida Y, Kishida Y Fau - Hosokawa M, et al. Incidence and risk factors of postoperative delirium in cervical spine surgery. (1528-1159 (Electronic)).

45. Vidan M, Serra JA, Moreno C, et al. Efficacy of a comprehensive geriatric intervention in older patients hospitalized for hip fracture: a randomized, controlled trial. Journal of the American Geriatrics Society 2005;**53**(9):1476-82.

46. Vollmer C, Rich C Fau - Robinson S, Robinson S. How to prevent delirium: a practical protocol. (0360-4039 (Print)).

47. Wanich Ck Fau - Sullivan-Marx EM, Sullivan-Marx Em Fau - Gottlieb GL, Gottlieb Gl Fau - Johnson JC, et al. Functional status outcomes of a nursing intervention in hospitalized elderly. (0743-5150 (Print)).
